# Comparative toxicity study of three surface-modified titanium dioxide nanoparticles following subacute inhalation

**DOI:** 10.1186/s12989-025-00620-1

**Published:** 2025-02-24

**Authors:** Dirk Schaudien, Tanja Hansen, Thomas Tillmann, Gerd Pohlmann, Heiko Kock, Otto Creutzenberg

**Affiliations:** Fraunhofer Institute for Toxicology and Experimental Medicine ITEM, Hannover, Germany

**Keywords:** Nanotoxicology, Surface modification, Titanium dioxide, Occupational exposure scenario, Histopathology

## Abstract

**Background:**

This study aimed to compare the toxic effects of three different titanium dioxide nanoparticles encoded in the European nanomaterial repository as NM-103 (rutile, hydrophobic), NM-104 (rutile, hydrophilic), and NM-105 (anatase/rutile, hydrophilic), suggesting different toxic potentials after uptake in the lungs. Wistar rats were exposed by nose-only inhalation to aerosol concentrations of 3, 12 and 48 mg/m^3^ for 4 weeks. This dosing scheme should induce non, partial and complete lung overload. The 4-week inhalation period was followed by 3-, 45- and 94-day exposure-free periods. Investigations according to the OECD 412 guideline were performed. Additional examinations, such as transmission electron microscopy and image analysis of tissue slides and cytospots, were performed to reveal possible differences among the three particle types.

**Results:**

Bronchoalveolar lavage fluid from the groups exposed to low concentrations of NM-103 or NM-104 presented slight inflammation. In the mid- and high-exposure groups, this was also present for the NM-105 group, however, weaker than those of NM-103 and NM-104.

Histologically, all three groups presented similar distributions of particles in the respiratory tract. Although marginal differences in the degree of some changes exist, no obvious differences in the degree or characteristics of the induced lesions were observable. In general, compared with the higher exposure groups, all the middle exposure groups presented a greater accumulation and aggregation of macrophages at the terminal bronchi. Using transmission electron microscopy, particles were detected mainly in intraalveolar macrophages, followed by type 1 pneumocytes in the low- and mid-concentration groups and intraalveolar free particles in the high-concentration groups. Compared with the other groups, the NM-103 group presented greater numbers of free particles in the alveoli and fewer in the macrophages.

With image analysis, the movement of particles to the bronchus-associated lymphoid tissue and lymph nodes could be detected comparably for the three different particle types.

**Conclusions:**

The no observed adverse effect concentration was 3 mg/m^3^ for all three different TiO_2_ particles. Despite minimal differences, a ranking mainly based on granulocyte influx into the lung was NM-104 > NM-103 > NM-105.

**Supplementary Information:**

The online version contains supplementary material available at 10.1186/s12989-025-00620-1.

## Background

Titanium dioxide is one of the most frequently commercialized nanoparticles worldwide. It is used as a white pigment in different products, such as dyes, sunscreens, fabrics, cosmetics and food [2]; [45]. Some TiO_2_ dusts often serve as negative particle controls in toxicological studies because their relative inertness leads to significant pulmonary changes only at severe overload doses [6, 17, 28]. Decades ago, it was reported that nanoscaled titanium dioxide exhibited increased specific toxicity compared with the microscaled fraction with the same chemistry [32, 42]. In addition, the retention time in the lungs was prolonged due to an increased access rate of particles to the interstitium [14].

The observed effects correlated with a surface-based dosing scheme rather than with a mass-based dosing scheme [35]. Therefore, the retained surface area (retained mass multiplied by the specific surface area; unit: m^2^) might better determine the toxicity outcome [36, 42]. More specifically, Cosnier showed for short- and long-term studies that the retained surface area correlates with the percentages of polymorphonuclear neutrophils in the bronchoalveolar lavage fluids in a dose-dependent way [9].

Höhr et al. [20] reported that, in an acute rat study, the surface area rather than the surface coating was the predominant determinant of the inflammatory response after intratracheal instillation of fine and ultrafine TiO_2_.

The size of agglomerates in the airborne environment also influences the aerodynamic diameter and, consequently, the location of deposition in the respiratory tract. Similarly, the severity of the inflammatory and cytotoxic reactions caused by TiO_2_ nanoparticles correlates with the status of agglomeration [31].

TiO_2_ nanoparticles occur mainly in two crystal structures: anatase, rutile and a combination of these two. The anatase crystal structure of TiO_2_ seems to be more toxic than the rutile crystal structure [51, 53, 54]. Modifications of the surface can clearly change the potential for lung toxicity [41], [47]. Warheit et al. [53] reported that in an inhalation toxicity study with differently coated hydrophilic TiO_2_ particles, only the variant with the highest amorphous silica content induced slight collagen deposition. In an intratracheal instillation test conducted by Oberdörster [36] in rats, nano-TiO_2_ functionalized with hydrophobic silane induced a stronger response than did coating with the uncoated hydrophilic particles. In general, the incorporation of TiO_2_ nanoparticles in an aged paint matrix considerably reduces inflammogenicity in the lungs Thoustrup [41, 47].

Differences in the specific surface of nanomaterial samples occur due to variations in the mean particle diameter, e.g., 5, 20 and 50 nm, or in the production and aging process following the generation of primary particles, including functionalization with physisorbed organics or octylsilane (chemical bonds) [56]. In addition, chemical surface modifications of nanoparticles are often performed to optimize them for technical applications, e.g., for better integration into polymer matrices. When both surfaces, the nanoparticle and the polymer, harmonize physicochemically well with each other, better mechanical enforcement can be achieved than passive interactions [16]. However, such modifications might also evoke changes in toxicity. Physicochemical characteristics influence the toxicity of nanoparticles [45]; [7]. Hydrophobic nanoparticles disperse harder in alveolar fluid but show an enhanced ability to penetrate into cell membranes and nuclear pores [29]. Compared with neutral or negatively charged nanoparticles, positively charged nanoparticles are more easily taken up by cells and can disrupt lysosomes to induce cell death [3, 45, 60].

Some studies comparing different TiO_2_ particle modifications in intratracheal instillation or inhalation tests have been performed [4, 5, 54]. However, no set of three particles with varied surface chemistry and crystalline structure has been investigated systematically in a 28-day inhalation test focusing on nanoparticle-specific endpoints so far.

In this study, we aimed to compare three types of TiO_2_ nanoparticles with various surface chemistries and crystalline structures under the same aerosol conditions in a 28-day inhalation toxicity test. Among physico-chemical properties, the specific surface area (SSA) is a central determinant in particle toxicology; to avoid an interfering influence of this parameter and to set the focus on surface functionalization similar SSA values were chosen for the set of three particles.

## Methods

### Experimental design of the study

**Fig. 1:**
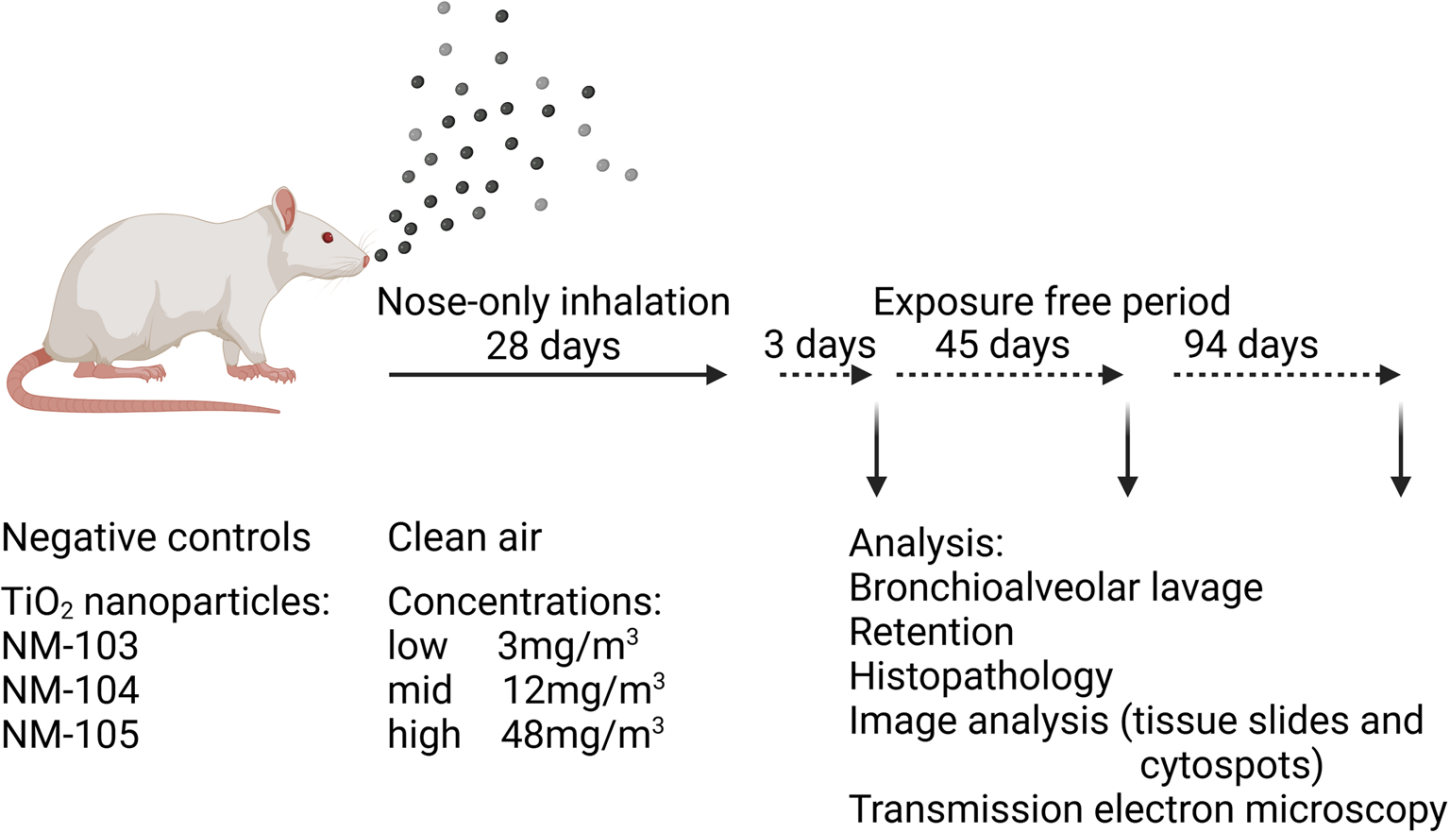
Study overview. The different test articles, exposure concentrations, time points and endpoints are shown. This figure was created in BioRender.com

Coded samples ordered from the Joint Research Centre (European Commission, JRC Nanomaterial Repository) in Ispra (Italy) were selected (Fig. 1), as this repository makes it easy for any laboratory to obtain identical test material, including a systematically documented physicochemical characterization. The objectives of this 28-day inhalation toxicity study with three TiO_2_ varieties were:

- To mimic an occupational exposure scenario by using a dry dispersion technique for aerosolizationto use a dosing scheme resulting in a non-overload, partial overload and complete lung overload situation in the low, mid and high aerosol concentration groups, respectively
- To examine the inflammogenicity of the three test items
- To analyze the toxicokinetic fate of TiO_2_ agglomerates following deposition in lungs
- To identify the respiratory cell types responsible for the uptake of these particles

The endpoints investigated upon cessation of exposure after 3, 45 and 94 days of the exposure-free period were according to OECD guideline 412: bronchoalveolar lavage fluid (BALF) analysis, histology, and chemical analysis of the lung, liver and brain. In addition,transmission electron microscopy (TEM) and image analysis of lung and lymph node histology slides and bronchoalveolar lavage cytospots were included to investigate possible differences in distribution behavior.

### Dosing scheme

The concept of lung overload was substantially developed by Morrow [27] who investigated the clearance capacity of the alveolar macrophage compartment in the deep lungs of rats. He focused on the dose-dependent overfilling of alveolar macrophages by engulfment and uptake of low soluble particles,consequently, a reduction of the macrophage motility (so-called volumetric overload effect) was observed. Oberdörster [34] emphasized the pivotal role of particle volume and assessed that lung overload is reached at a threshold lung burden of 1 µl/g rat lung tissue.

This experimental design of the study was harmonized with the German Federal Institute for Occupational Safety and Health (BAuA). The dosing scheme intended to achieve an unimpaired clearance capability at physiologically controllable lung burdens (in the low dose group) as well as a partial and a complete lung overload with the corresponding retardation of lung clearance (in the mid and high dose groups, respectively). Under overload conditions, the particle burden is not fully engulfed by the macrophage pool and free particles are present in the alveolar lumen. The toxicokinetic fate of TiO_2_ agglomerates, i.e. a potential translocation of nanoscaled particles from the lungs to remote organs, if existing at all, can be elucidated at higher probability. In addition, the presence of free particles can facilitate the differentiation of respiratory cell types responsible for the uptake of these particles.

The aerosol concentrations used in this animal experiment to induce a slight and a significant lung overload do not reflect actual exposure scenarios at the workplace.

### Characterization of test items

A TiO_2_ set of three particles was chosen from the EU Commission/Joint Research Centre (JRC, [38]), which varied in their surface and crystallinity. These particles had already been characterized by the Joint Research Centre and are supplied to laboratories to provide accepted and validated test items of the same identity to everyone. Overview photographs of the particles are presented in Supplemental Fig. 1, which shows agglomerated aggregates of µm size as well as partially individual nanosized aggregates. The particle size distributions of the bulk materials were measured by scanning electron microscopy at the JRC [38]. Additionally, the specific surface area of the test samples was determined via Fraunhofer IKTS (Dresden, Germany) using the Brunauer–Emmett–Teller (BET) method [8]. The physicochemical properties of these materials are summarized in Table 1.

**Table 1:** Characteristics of the test items

EU/JRC-code	PPD (nm)	Surface - character - modified with - specific surface (m^2^/g)	Name	Crystal type
NM-103	20	Hydrophobic Coated with alumina and dimethicone (Silicone) 60^a^ (56.2)^b^	UV TITAN M212	Rutile
NM-104	20	Hydrophilic Coated with alumina and glycerol 60^a^ (46.0)^b^	UV TITAN M262	Rutile
NM-105	22	Hydrophilic Not coated 60^a^ (56.3)^b^	TiO_2_ P25 Commercial sample, characterized by [38]	Anatase/Rutile 80%/20%

PPD: Primary particle diameter; Specific surface^a^ given by producer^b^ analysis by Fraunhofer IKTS, Dresden, Germany

Dimethicone = PDMS. The chemical formula for PDMS is CH_3_[Si(CH_3_)_2_O]_*n*_Si(CH_3_)_3_, where *n* is the number of repeating monomer [SiO(CH_3_)_2_] units. Industrial synthesis can begin from dimethylchlorosilane and water via the following net reaction: *n* Si(CH_3_)_2_Cl_2_ + *n* H_2_O → [Si(CH_3_)_2_O]_*n*_ + 2*n* HCl

NM-103 and NM-104 underwent surface finishing with alumina. For NM-103, a polysiloxane polymer layer is also involved. The overall surface treatment mass was approximately 2% each. For details, see [38]

### Animals

Male Wistar rats [strain Crl:WI (Han)] were purchased from Charles River Sulzfeld (Sulzfeld, Germany). The animals arrived at the institute approximately 5 weeks old and were housed for one week to acclimatize to the animal house conditions. As diet a commercial chow in pellet form was used (ssniff "V1534"; sniff Spezialdiaeten GmbH, Soest, Germany). Tap water from the Hannover city water supplier (Stadtwerke Hannover, Germany) as well as the diet were offered ad libitum. For a period of 3 weeks prior to exposure, the animals were trained to become accustomed to nose-only tubes. The age of the animals at the start of exposure was approximately 9 weeks, and the weights were approximately 250 g. The rats were exposed by nose-only inhalation. A total of 360 male rats were used for this study (approval file # 33.9–42,502-04–11/0519/LAVES, Oldenburg, Lower Saxony).

### Nose-only inhalation

Air flow, temperature and relative humidity were measured continuously and recorded by 20-min means. The limits were set at 22 ± 2 °C for temperature and 55 ± 15% for relative humidity.

Rats were exposed to aerosol concentrations of 3 (low), 12 (mid) and 48 (high) mg/m^3^ for 28 days (6 h/day, 5 days/week), whereas concurrent controls inhaled clean air (Fig. 1). This dosing scheme resulted from calculation of the expected particle lung burdens and was aimed at inducing non, partial overload and significant overload conditions in the low, mid and high exposure groups, respectively. The endpoints were subsequently analyzed after an additional exposure-free period of 3, 45 and 94 days (Fig. 1, supplement file 1).

The particulate sample aerosols were generated via a dry dispersion technique with pressurized air. Dispersion was achieved by a feeding system and a high-pressure, high-velocity pressurized air dispersion nozzle developed by Fraunhofer ITEM [24]. The disperser was fed with the test items under computerized control, i.e. with a feedback loop to the actual aerosol concentrations measured by an aerosol photometer. The photometer gives a scattering light signal which is proportional to the particle concentration, if the particle size distribution is constant. The ratio between photometer signal and concentration was determined throughout the study by comparing to gravimetric concentrations. Filter samples were collected daily for each exposure unit to record the gravimetrical aerosol concentrations.

The mass median aerodynamic diameter (MMAD) was measured using a Marple™ cascade impactor; the values ranged from 0.59 to 1.57 µm, thus demonstrating the high respirability of the aerosols. The results are presented in Table 2.

**Table 2:** Gravimetrical aerosol concentrations and mass median aerodynamic diameter (MMAD)

Target concentration (mg/m^3^)	NM-103	NM-104	NM-105	NM-103	NM-104	NM-105	NM-103	NM-104	NM-105
	low 3			mid 12			high 48		
*Aerosol concentrations (mg/m*^*3*^*)*									
Mean ± SD N = 24	3.06 ± 0.18	3.22 ± 0.40	3.17 ± 0.21	12.13 ± 0.25	12.23 ± 1.48	12.31 ± 1.62	49.70 ± 8.20	47.70 ± 7.60	47.70 ± 4.80
*MMAD (µm)*									
Mean	1.17	1.00	0.62	1.03	0.91	0.83	0.62	1.57	0.59
GSD	2.92	3.94	4.79	2.64	3.26	4.02	4.27	3.50	6.19
N	2	2	2	2	2	3	3	3	3

MMAD: mass median aerodynamic diameter; GSD: geometric standard deviation

According to the Multiple-Path Particle Dosimetry (MPPD; v 3.04) model [1], with an MMAD of approximately 1 µm, a GSD of 3 and an agglomerate density ρ_Agg_ = approximately 1.6 [37], the retained particle masses would result in approximately 0.3, 1.1 and 4.4 mg/lung for the low, mid and high exposure concentrations (3, 12, 48 mg/m^3^), respectively (calculated deposition fraction = 6.4%).

### Body weights and organ weights upon necropsy

Body weight data were recorded throughout the study. During necropsy, the terminal body weights were measured, organs such as the lungs, liver, kidneys, adrenals, testes, epididymides, thymus, spleen, brain, and heart were trimmed, and the wet weights were recorded.

### Bronchoalveolar lavage (BAL)

Bronchoalveolar lavage was performed on 6 rats per group 3, 45 and 94 days after the end of exposure. The method of Henderson et al. [18] was used with minor modifications.

Following preparation, the left lung lobe was lavaged with saline using two lavages of 2.5 ml each. The first lavage fluid sample was collected for differential cell counting and enzyme/total protein analysis. A second series of 3 lavages, each with 2.5 ml and including gentle lung tissue massage, followed. The combined lavages were used for the analysis of reactive oxygen intermediates (ROIs) generated by alveolar leukocytes.

The leukocyte concentration of the bronchoalveolar lavage fluid was determined via a counting chamber, and two cytoslides, each with two cytospots were prepared using a cytocentrifuge (Shandon Co., Frankfurt, Germany) for differential cell counting (macrophages, neutrophils, eosinophils, and lymphocytes). On each slide, 2 × 100 leukocytes were differentiated twice, and the means of a total of 4 evaluations were calculated.

After centrifugation of the lavage fluid, the following biochemical indicators relevant for the diagnosis of lung toxicity were determined in the supernatant: lactic dehydrogenase (LDH) (cytotoxicity: release of cytosolic LDH, β-glucuronidase (damage to macrophages), and total protein (transudation)). These parameters were analyzed according to routine clinical chemistry protocols using a Cobas Fara device (Roche Co., Grenzach, Germany).

The reactive oxygen intermediate (ROI) production by alveolar macrophages was measured via chemiluminescence. The measurements were performed via MicroLumate (Berthold Technologies, Bad Wildbad, Germany) to measure lucigenin-mediated chemiluminescence. For this purpose, 2 × 10^5^ cells/100 µl were pipetted into the wells of a microtiter plate in culture medium. 8 wells were used for every animal, representing the quadruplicates for the medium control and the stimulated samples. After pipetting the cells, 10 µl of lucigenin was added to each well, and the plate was measured via MicroLumate to determine the background level of the cells. After that measurement, 10 µl of zymosan as a phagocytic trigger or culture medium was added to the respective wells, and ROI production was measured again for 30 min at 37 °C using MicroLumate. The cells were recovered via centrifugation (256 × g for 10 min at 4 °C) and resuspended in RPMI (containing 10 mM HEPES). Cell counts and viability were assessed in a CASY device (Schärfe System GmbH, Reutlingen, Germany).

### Retention of test items in lungs

After sacrifice, the right lung lobes (from 6 animals per group) were cut into smaller pieces and subjected to lyophilization and subsequent low-temperature ashing. The remaining ash was put into 25 ml water (Milli-Q) and shaken until homogeneous. After a further 30-min period the particle suspension was filtrated using Nucleopore™ filters with pore size 0.2 µm (Whatman Co., USA). This pore size is able to separate almost completely the particulate matter from ionic titanium. Afterwards, the filter was rinsed with 25 ml additional water. The test items were analyzed via ion-coupled plasma mass spectrometry (ICP-MS; X Series 2, ThermoFisherScientific™). Recoveries: Plasma ashing—Ionic Ti: 103–105%; Filtration—Ionic Ti: 96%; Chemical analysis—QC standards: 101%; NBS SRM 349: 102%—LoQ: 10 ppb.

In addition, particle retention was determined in exemplary organs, such as the liver and brain.

The retention half-times in lungs were calculated assuming a first order kinetics (m = m_0_ x e^−kt^). An exponential curve fit was processed based on the individual retention data at day 3, 45, 94 post-exposure (3 time-points; 6 animals each) in order to calculate the clearance coefficient and the half-time: k = ln2/t_1/2_ (Statistica™ software).

### Histopathology

Histopathology was performed in 6 animals per group at the indicated time points (3, 45, and 94 days after the end of exposure). Full histopathology was conducted on the respiratory tract and other organs and tissues, as listed in OECD guideline 412 “Subacute Inhalation Toxicity: 28-Day Study”, of all animals in the clean air control group and the high-exposure-concentration groups. Histopathology of the respiratory tract, including the left lung lobe, lung-associated lymph nodes, trachea, larynx, pharynx, nasal cavities and visceral pleura, was performed for all the animals in the low- and mid-exposure groups. The trimming was performed according to [39], [23] and [26]. The tissues were examined histopathologically by a board-certified veterinary pathologist. For the evaluation of the lung and lung-associated lymph nodes, a previously developed scheme was used (supplement file 2, [56]).

Tissues were fixed in 10% buffered formalin, embedded in paraffin. The lung was fixed by installation at a pressure of 25 cm of water. Three µm thick sections were retrieved from the paraffin blocks and subsequently stained with hematoxylin and eosin (HE). To investigate possible fibrogenesis or fibrosis, an additional slide of the left lung lobe was stained with Masson’s trichrome special stain.

#### Image analysis of tissue slides and cytospots

To detect possible differences between the three TiO_2_ particles, the amount of TiO_2_ particles was also measured in histologic lung and lung-associated lymph node slides. HE-stained slides were digitized via a Mirax slide scanner (3dHistech, Hungary). The areas of the TiO_2_ particles in the tissue slides were measured by thresholding via Visiopharm software (Visiopharm A/S Hørsholm, Denmark) as previously described [43]. For the measurement of the amount of TiO_2_ in the bronchus-associated lymphoid tissue (BALT) in the lung, those tissue areas were annotated prior to measurement. Animals whose BALT tissue was not visible on the slide were excluded from the analysis.

To determine whether differences in the particle load of the cells were caused by the different TiO_2_, cells in the bronchoalveolar lavage were analyzed. The two cytospots used for differential cell counting of the bronchoalveolar lavage fluid were digitized via the NanoZoomer S210 (Hamamatsu Photonics, Herrsching am Ammersee, Germany). An algorithm was developed to identify TiO_2_ particle-laden cells via Visiopharm software, and positive and negative cells were counted. Furthermore, the particle load of the cells was classified into three categories: low particle load (1 +), medium particle load (2 +) and high particle load (3 +). The positive cells were allocated to the three categories via Visiopharm software. The percentage of particle-laden cells among all the cells in the cytospot as well as the percentage of the cells with a high particle load among the particle-laden cells were calculated.

#### Transmission electron microscopy

At the respective time points to be investigated (exposure-free days 3, 45 and 94), the right part of the lung, including the right cranial, right middle and right caudal lung lobes as well as the accessory lung lobe, was fixed by instillation of 5% glutaraldehyde solution (pH 7.2) for at least 24 h. Following fixation of the tissue, the volume of the fixed tissue was determined via the method of Scherle [42]. To avoid biased sampling, multiple samples per organ were taken via the *systematic uniform random sampling* method as described previously [13, 21]. The samples were further processed for transmission electron microscopy as previously described [10]. A total of 55,000 square micrometers of three randomly chosen samples per animal were investigated at a magnification of 10,000 × with a Zeiss TEM Leo 912. The amount and location of the nanoparticles found were noted and assigned to the compartments. The compartments in which the nanoparticles were detected were defined as follows: intraalveolar macrophages, free within the alveoli, pneumocyte type I, and interstitium.

#### Statistics

Differences between groups were considered statistically significant at *p* < 0.05. Organ weights and bronchoalveolar lavage were recorded in the Laboratory Information Management Software Provantis version 8.4.3.1 (Instem®, UK) and initial statistical analysis was done comparing against the respective negative control group using the Dunnett's test.

Further analyses were done with the Statistica software (Version 13, StatSoft GmbH, Hamburg, Germany) using analysis of variance (ANOVA), for an initial multifactorial analysis, or students t-test, for group-wise comparison, and reactive oxygen intermediates data against the respective negative control. For the direct comparison of TiO_2_ treated groups among themselves, control animals were excluded from that analysis.

## Results

All the animals survived the exposure period of the study and tolerated the exposure well in all the concentration groups. No clinical observations outside the normal limits were recorded, and body weights were not significantly different between the different groups. Additionally, the wet weights of organs obtained at necropsy, including the liver, kidneys, adrenals, testes, epididymides, thymus, spleen, brain, and heart, were not significantly different to the concurrent negative control group.

### Absolute and relative wet lung weights

The absolute and relative wet weights of the lungs of all three TiO_2_-treated groups increased in a concentration-dependent manner (statistically significant in the mid-exposure concentration groups of NM-103 for absolute and NM-103, 104 and 105 for relative lung weight, and statistically significant in all high-exposure concentration groups for absolute and relative lung weights). After an exposure-free period of 94 days, the absolute and relative lung weights in the mid-exposure concentration groups were comparable to control levels, whereas the absolute and relative lung weights in the high-exposure concentration groups were still significantly increased (see supplemental Fig. 2).

**Fig. 2:**
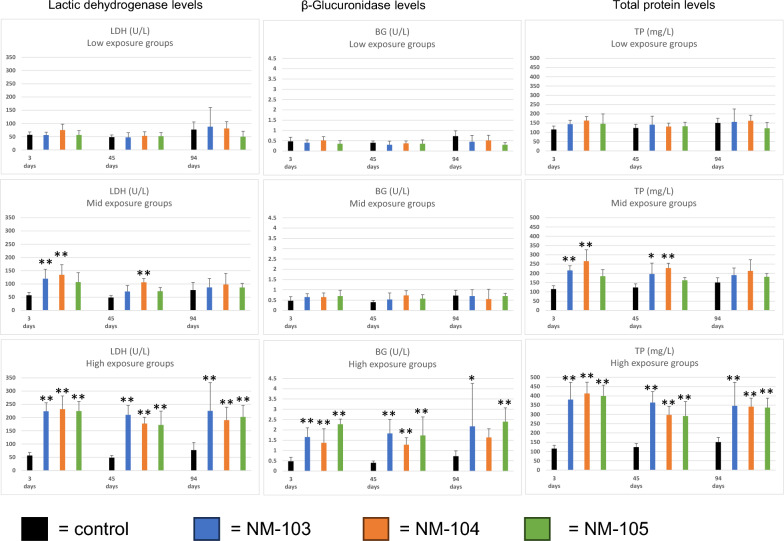
Results of the bronchioalveolar lavage analysis. Results of lactic dehydrogenase (LDH), β-Glucuronidase (GB) and total protein (TP) are given of the low, mid and high exposure groups. * indicate significant differences (*P* < .05), ** indicate (*P* < .01), *** indicate (*P* < .001) to the respective negative control group

### Analysis of bronchoalveolar lavage (BAL) fluid

#### Lactic dehydrogenase, β-glucuronidase and total protein

None of the three different TiO_2_ low-exposure groups showed elevated levels of the investigated parameters lactic dehydrogenase, β-glucuronidase or total protein to the concurrent control groups.

The groups exposed to mid concentrations of NM-103 and NM-104 presented statistically significant increases in lactic dehydrogenase (LDH) and total protein levels on postexposure day 3 (Fig. 2) to the concurrent control groups. After an exposure-free period of 45 days, significant levels persisted only in the NM-104 mid exposure-concentration group for LDH and total protein, whereas in the NM-103 mid exposure-concentration group, only total protein levels were significantly increased to the concurrent control groups. After 94 days of exposure free time, all the mid exposure concentration groups were comparable to the concurrent control groups. For NM-105, no statistically significant difference to the control was observed for any parameter of the mid exposure-concentration group.

All the high-exposure-concentration groups presented significant increases in the three endpoints up to an exposure-free period of 94 days, with the exception of β-glucuronidase in the NM-104 group after 94 days.

In general, the statistical analysis (ANOVA) revealed a statistically significant influence of the exposure levels (3, 12 and 48 mg/m^3^) on all three measured parameters (*p* < 0.01).

In the comparison of the three different TiO_2_, there were a significant differences in total protein values between the mid-exposure-concentration groups of NM-104 and NM-105 at day 3 (*p* < 0.05) and day 45 (< 0.001), in LDH at day 45 between the mid-exposure-concentration groups of NM-103 and NM-104 (*p* < 0.05) and NM-104 and NM-105 (*p* < 0.01), and in β-glucuronidase between the high-exposure-concentration groups of NM-103 and NM-105 (*p* < 0.05) and NM-104 and NM-105 (*p* < 0.05).

### Differential cell counts

**Fig. 3:**
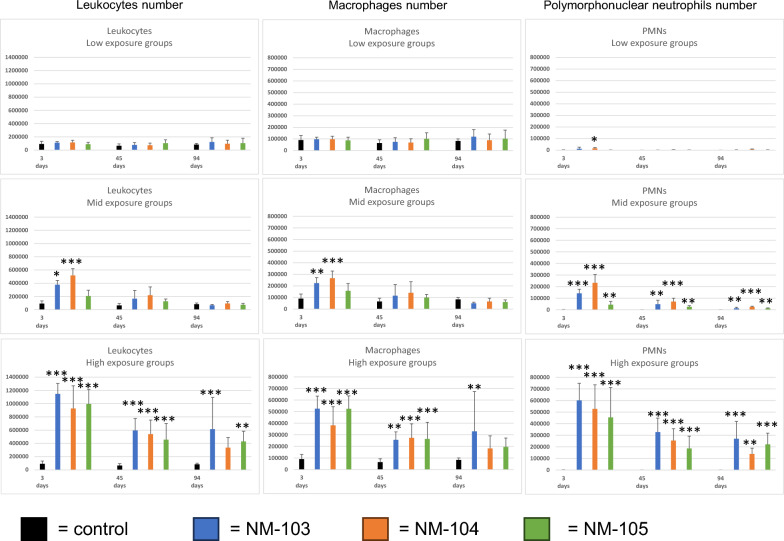
Results of the bronchioalveolar lavage analysis. Results of leukocytes, macrophages and polymorphonuclear neutrophil (PMN) are given of the low, mid and high exposure groups. * indicate significant differences (*P* < .05), ** indicate (*P* < .01), *** indicate (*P* < .001) to the respective negative control group

Leukocyte cell concentrations were significantly increased after 3 days of exposure free time in the NM-103 and NM-104 mid exposure groups (Fig. 3, supplement file 3) but not in the NM-105 group. After 45 and 94 days of exposure free time, the values were comparable to the concurrent control group. In all three high-exposure groups, a strong increase was observed after 3 days, which remained statistically significant until day 94.

In the direct comparison of the leucocyte number due to inhalation of the different TiO_2_, ANOVA analysis showed statistically significant differences only at day 3 in the mid exposure groups (*p* < 0.01). The groups-wise comparison revealed significant differences between NM-103 and NM-104 (*p* < 0.05), NM-103 and NM-105 (*p* < 0.01), and NM-104 and NM-105 (*p* < 0.001).

In the low-exposure-concentration groups, NM-105 resulted in a PMN number similar to clean air control groups, whereas NM-103 and NM-104 resulted in slight inflammation with up to 10% PMN (see supplement Fig. 3, supplement file 3). After 45 and 94 days of exposure free time, only the animals exposed to low concentration of NM-104showed elevated, but not statistically significantly increased, neutrophilic levels ranging from 5 to 8%.

In the mid and high-exposure-concentrations groups, the PMNs were significantly elevated in all three TiO_2_ at all time points.

In the comparison of the three different TiO_2_ concerning the PMNs values, there were a significant differences in the low-exposure-concentration groups between NM-104 and NM-105 (*p* < 0.01) at day 3, in the mid-exposure-concentration groups at day 3 between NM-103 and NM-104 (*p* < 0.05), NM-103 and NM-105 (*p* < 0.001), and NM-104 and NM-105 (*p* < 0.001), in the mid-exposure-concentration groups at day 45 between NM-104 and NM-105 (*p* < 0.01), and in the mid-exposure-concentration groups at day 94 between NM-103 and NM-104 (*p* < 0.05), and NM-104 and NM-105 (*p* < 0.01). In the high-exposure groups no statistical differences were observed between the three different TiO_2_ groups at any time point.

In general, the statistical analysis (ANOVA) revealed a statistically significant influence of the exposure levels (3, 12 and 48 mg/m^3^) on the differential cell counts (*p* < 0.01).

In conclusion, the NM-105 treated animals exhibited especially in the mid-exposure-concentration compared with the NM-103 and NM-104 groups, a weaker inflammatory reactions as seen by lower numbers of leucocytes and PMN in the bronchoalveolar lavage fluid (Fig. 3).

### Reactive oxygen intermediates

Stimulation with zymosan prominently induced the secretion of reactive oxygen intermediates (ROIs) by alveolar macrophages in all groups, including the control group.

The results on day 3, including zymosan activation, revealed significantly increased ROI secretions in the mid and high-exposure-concentration groups of only NM-104 but not NM-103 and NM-105. Following exposure-free periods of 45 and 94 days, no significant increase was observed. (supplement file 4).

### Retention of test items in targets and other organs

The assumptions for the model calculations (MPPD model v 3.04; Anjilvel and Ashgarian, 1995; National Institute RIVM, 2002) were as follows:

Agglomerate density: 1.6 g/cm^3^; deposition only.

Unisex: Minute volume: 0.2 ml/min;

Agglomerate density ρAgg = approx. 1.6 g/cm^3^ (measured by pycnometry; [37]),

MMAD: approx. 1 µm (= mean value);

Deposition only.

Regional deposition fraction in deep lung: 0.064.

Retained mass in lungs: 0.2 l/min × 360 min × 20 days × 48 mg/m^3^ × 6.4% = approx. 4.4 mg/rat.

The predicted retention were approximately 0.3–1.1–4.4 mg/lung for the TiO_2_ low, mid and high exposure concentration groups, respectively, at day 1 post-exposure.

**Fig. 4:**
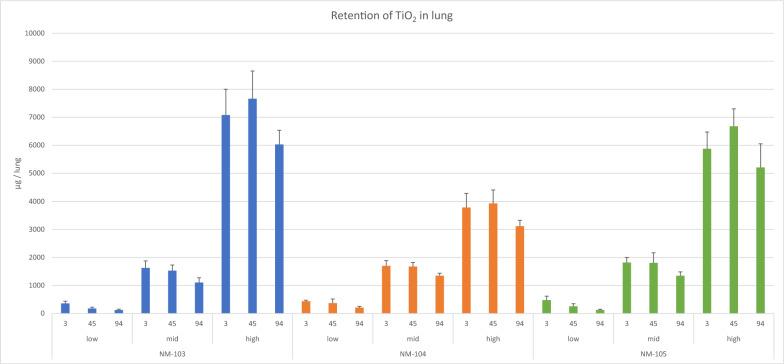
Results of the retention measurement of the lung. Results are given of the low, mid and high exposure groups and the different exposure free days (3, 45, 94)

Actual retention measured: In the low exposure groups, 0.4, 0.4 and 0.5 mg/lung, in the mid exposure concentration groups 1.6, 1.7 and 1.8 mg/lung, and in the high exposure concentration groups 7.0, 3.8 and 5.9 mg/lung were determined for NM-103, NM-104 and NM-105 at day 3 post-exposure, respectively (Fig. 4, supplement file 5). The actually retained masses correspond quite well to the theoretical data derived from the model and show a high similarity in the groups of the same exposure concentration, with one exception: an underproportionally lower mass was detected in the NM-104 high exposure concentration group, which could be explained by a higher MMAD value in this group (1.6 µm, see Table 2) leading to an increased tendency to build agglomerates in the aerosol compared to NM-103 and NM-105 (MMADs: approx. 0.6 µm) and, therefore, to a lower deposition fraction.

During the exposure-free period, a significantly retarded lung clearance was observed in the high-exposure-concentration groups. In contrast, in the mid exposure-concentration group, a partially retarded clearance was detected, and in the low-exposure-concentration groups, an unaffected, physiological lung clearance (approximately 60 days in rats) can be concluded (Table 3). This reflects the different degrees of clearance retardation due to the various lung loads.

**Table 3:** Retention half-times during a 94-day exposure-free period

Half-times (days)	NM-103	NM-104	NM-105
Low exposure concentration	59	85	46
Mid exposure concentration	162	267	204
High exposure concentration	373	315	485

Exponential curve fit (Statistica™ software) assuming a first order kinetics

The soluble moiety of the test items in the lungs reached up to 5.5% of the total mass in the low-exposure-concentration groups; however, it was not more than 2.2% and 0.9% in the mid and high-exposure-concentration groups, respectively (supplement file 5). These results suggest that the solubility of the test material is limited by a given maximum under conditions of lung ambience.

In the liver, the detected amounts of TiO_2_ were generally below the limit of detection. However, some rats showed considerable masses of particulate test items. Approximately 14 µg/liver was detected in two rats in the NM-104 mid exposure concentration group, and even 206 µg/liver was detected in the NM-104 high exposure concentration group on day 94. Approximately 16 µg/liver on day 45 and 67 µg/liver on day 94 were used, each in 1 rat in the NM-105 high-exposure-concentration group (supplement file 6).

In the brain, the detected amounts of TiO_2_ were generally below the limit of detection (supplement file 6).

### Pathology

The macroscopic findings observed upon necropsy at the scheduled time points revealed that exposure had concentration-dependent effects on the lungs and lung-associated lymph nodes. Findings in the lungs, such as white discolored areas in the high-exposure-concentration groups, reflected the exposure concentration-dependent retention of test items. Furthermore, the lung-associated lymph nodes (LALNs) were slightly enlarged in most rats in the mid exposure concentration groups and moderately to severely enlarged in the high-exposure concentration groups.

Histologically, findings related to the inhalation of the TiO_2_ nanoparticles were observed in the lung, trachea, nasal cavity, larynx and LALN.

**Fig. 5:**
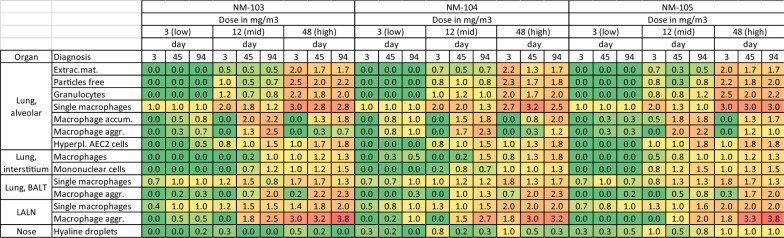
Presentation of data from findings in lungs, lymph node and nose related to the inhalation of the TiO_2_ particles. Numbers reflect the group mean of histopathologic investigation calculated by the sum of the individual severity grades of the animals of the respective group divided by the total number of animals of the respective group (n = 6). The different colors reflect additionally the severity grade of the diagnoses ranging from green (severity grade 0) to red (severity grade 4). Abbreviations: accum.: accumulation, aggr.: aggregation, BALT: bronchus-associated lymphoid tissue, extrac. mat.: extracellular material, hyperpl.: hyperplasia

**Fig. 6:**
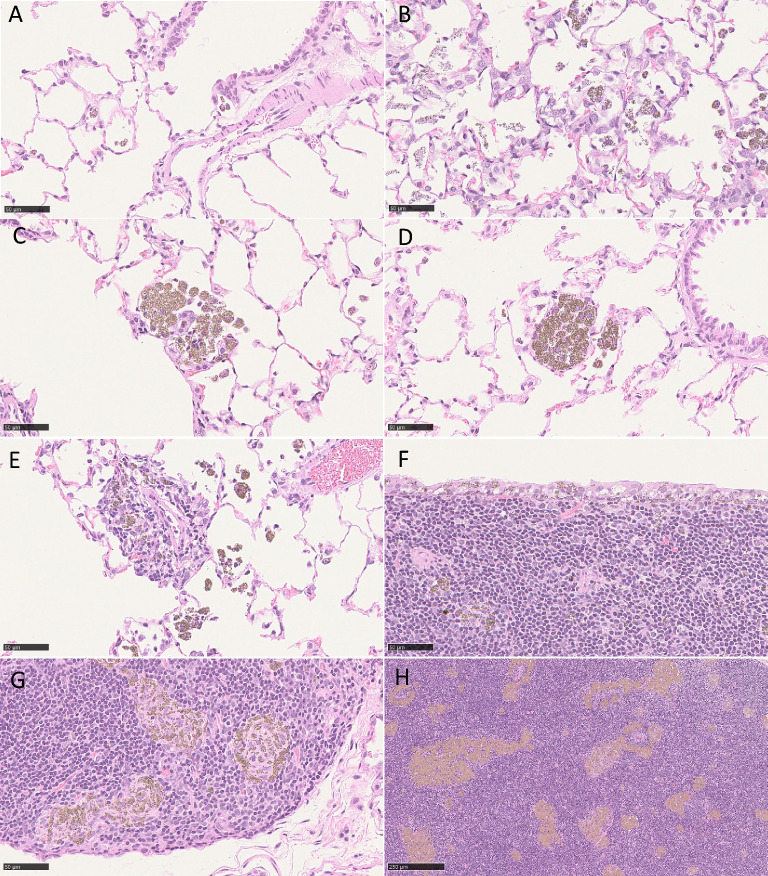
Exemplary images of the histopathologic lesions. **(A)** Single particle-laden macrophages in alveoli. Low NM-103 exposure, 3 days **(B)** rupture of macrophages with release of previously phagocyted particles. Alveolar infiltration by neutrophilic granulocytes. High NM-104, 3 days. **(C)** Macrophage accumulation. Mid NM-105 exposure, 45 days. **(D)** Macrophage aggregation. Mid NM-104 exposure, 94 days. **(E)** Interstitial particle-laden macrophages. High NM-104 exposure, 94 days **(F)** single particle-laden macrophages in bronchus-associated lymphoid tissue and intraepithelial particles. High NM-103 exposure, 3 days **(G)** Macrophages aggregation in bronchus-associated lymphoid tissue. Mid NM-103 exposure, 94 days **(H)** Macrophages aggregation in lung-associated lymph node. High NM-105 exposure, 94 days

Particle-laden macrophages were observed in a concentration-dependent manner in the lung, trachea, nasal cavity, larynx and LALN. In the lung, most of the particle-laden macrophages were located within the alveoli, with a minor portion in the bronchus-associated lymphoid tissue and in the interstitium (Figs. 5, 6; supplement file 7). At the end of the exposure-free period (94 days), the interstitial portion of the particle-laden macrophages was more prominent than at exposure-free day 3, indicating the time-dependent translocation of the particle-laden macrophages or the particles themselves from the alveoli into the interstitium. This is also reflected by a greater burden of particle-laden macrophages in bronchus-associated lymphoid tissue and lung-associated lymph nodes in animals after 94 exposure-free days. Furthermore, the distribution of intraalveolar particle-laden macrophages changed over time from an initially more disseminated distribution of single macrophages to a more multifocal distribution with loose macrophage accumulation and closer macrophage aggregation in the vicinity of the terminal bronchi (Fig. 6, supplement Figs 4, 5, and 6). Interestingly, macrophage accumulation and aggregation were more prominent in the mid exposure concentration group than in the high-exposure concentration group. The occurrence of particle-laden macrophages in the lung interstitium was accompanied in a concentration-dependent manner by interstitial mononuclear cell infiltration, which increased slightly over time. In addition, hyperplasia of alveolar epithelial cell (AEC) type 2 cells was observed mainly in the mid and high-exposure-concentration groups. Within the alveolar region, extracellular eosinophilic material accompanied by free particles within the alveoli and alveolar infiltration of granulocytes were observed in a concentration-dependent manner, which was still observable after 94 days of exposure free period. The detection of the previously mentioned lesions revealed an ongoing inflammatory stimulus in the higher exposure concentration groups. However, the development of a granulomatous inflammation or granuloma formation were not observed.

Moreover, particles were observed in an exposure concentration-dependent manner in the cytoplasm in the epithelium located above the bronchus-associated and nose-associated lymphoid tissue as well as at the carina of the trachea (Fig. 6), which is most likely due to direct impaction of the particles in that area. In the other parts of the respiratory tract, such as the larynx and trachea, single animals in the mid and high-exposure-concentration NM-103-, NM-104-, and NM-105-treated groups presented very slight subepithelial single particle-laden macrophages at exposure-free periods 3, 45, and 94 days.

In the nasal cavity, the observed changes were located mainly in the anterioventral compartment, which is most strongly exposed to airflow. The exposure concentration-dependent changes included eosinophilic cytoplasmic inclusions within the respiratory epithelial cells of the nasal septum and ventral nasal meatus. The occurrence of these eosinophilic inclusions (hyaline droplets) declined over time in most groups.

Apart from the respiratory tract, the other organs investigated did not show treatment-related changes. The data of the other organs investigated can be found in the underlying study report: https://www.baua.de/DE/Forschung/Forschungsprojekte/f2246.

All three different treatment groups (NM-103, NM-104 and NM-105) presented similar exposure concentration-dependent alterations. In addition, all three groups presented similar patterns of deposition and distribution of particles in the respiratory tract. Although slight differences in the degree of some changes exist, no obvious differences in the degree or characteristics of the induced lesions were observable histopathologically (Fig. 5).

### Image analysis of tissue slides

**Fig. 7:**
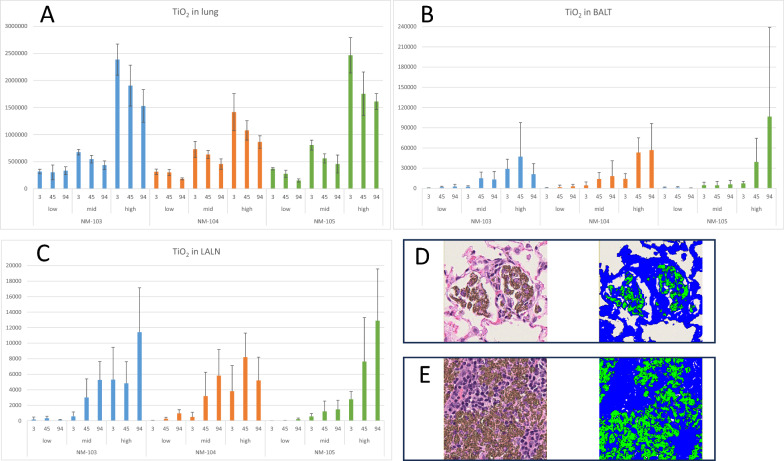
Results of the image analysis of the tissue slides. (**A**) Data of the lung tissue (**B**) Data of the bronchus-associated lymphoid tissue (BALT) tissue **(C)** Data of the lung-associated lymph node **(A-C)** Data is given of the low, mid and high exposure groups and the different exposure free days (3, 45, 94) **(D)** Exemplary image of the image analysis of the lung tissue, original (left) and analyzed (right) image **(E)** Exemplary image of the image analysis of the lung lymph node, original (left) and analyzed (right) image

Image analysis of the histological slides revealed a concentration-dependent increase in the amount of TiO_2_ in the lung (Fig. 7A; supplement file 8). In addition, a time-dependent reduction in TiO_2_ was observed during the exposure-free period. Slight differences occurred between the different TiO_2_ samples, especially in the high-exposure-concentration group is a statistically significant difference between NM-103 and NM-104 or NM-104 and NM-105 (always *p* < 0.01), with NM-104 showing a lower amount of TiO_2_, which is compatible with the retention analysis (Fig. 7A).

Focusing on bronchus-associated lymphoid tissue (BALT), an increase in TiO_2_ occurred during the exposure-free period, with an exposure concentration-dependent movement of TiO_2_ to the BALT (Fig. 7B). However, no statistically significant differences between the three different TiO_2_ materials were observed.

Similar to BALT, an increase in measured TiO_2_ occurred during the exposure-free period, with an exposure concentration dependency in the lung-associated lymph nodes (Fig. 7C). This is consistent with the movement of TiO_2_ to the lung lymph nodes. No statistically significant differences between the three different TiO_2_ materials were observed.

**Fig. 8:**
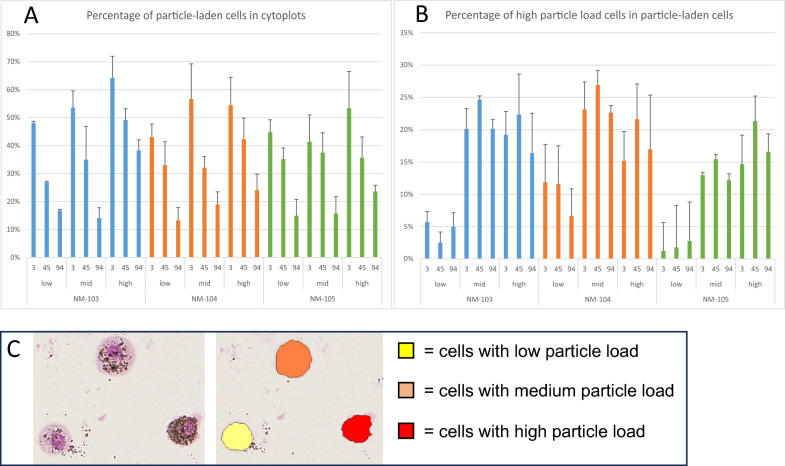
Results of the image analysis of the cytospots. (**A**) Percentage of particle-laden cells in all cells in the cytospots (**B**) Percentage of high particle load cells in particle-laden cells **(C)** Exemplary image of the image analysis of the cytspots, original (left) and analyzed and categorized cells (middle)

In the analysis of the cytospots, there was, in general, an exposure concentration-dependent increase in the number of particle-laden cells in the cytospots of the BAL fluid (Fig. 8A). This number decreases during the exposure-free period in a time-dependent manner. Interestingly, the percentage of cells with a high particle load among the particle-laden cells generally increased statistically significant (*p* < 0.01) from exposure-free days 3 to 45 and decreased again from exposure-free days 45 to 94 in the higher exposed groups (Fig. 8B). Compared with those in the NM-103 and NM-104 groups, the percentage of cells with a high particle load in the low and mid exposure groups of NM-105 was significantly lower (*p* < 0.001).

### Transmission electron microscopy analysis

Measurement of the lung volume at the different time points investigated (exposure free days 3, 45 and 94) revealed a statistically significant (*p* < 0.01) influence of the recovery day on the lung volume (see supplement Fig. 7). Consequently, the amount of TiO_2_ nanoparticles found per compartment was normalized to the measured lung volume. The following particles represent agglomerated nanoparticles as well as primary nanoparticles. Particles were counted when they were clearly distinguishable from other particles or surrounding structures as described by [13]. In the TiO_2_ nanoparticle treatment groups (NM-103, NM-104 and NM-105), the particles found consisted almost always of agglomerated primary nanoparticles (supplement Fig. 8). The particles, which were found free within the alveoli, were attached mainly to light electron-dense materials resembling surfactants or cellular debris (supplement Fig. 8), which is comparable to the extracellular eosinophilic material found histologically.

Within the negative control group, no electron-dense objects resembling TiO_2_ nanoparticles were detected.

**Fig. 9:**
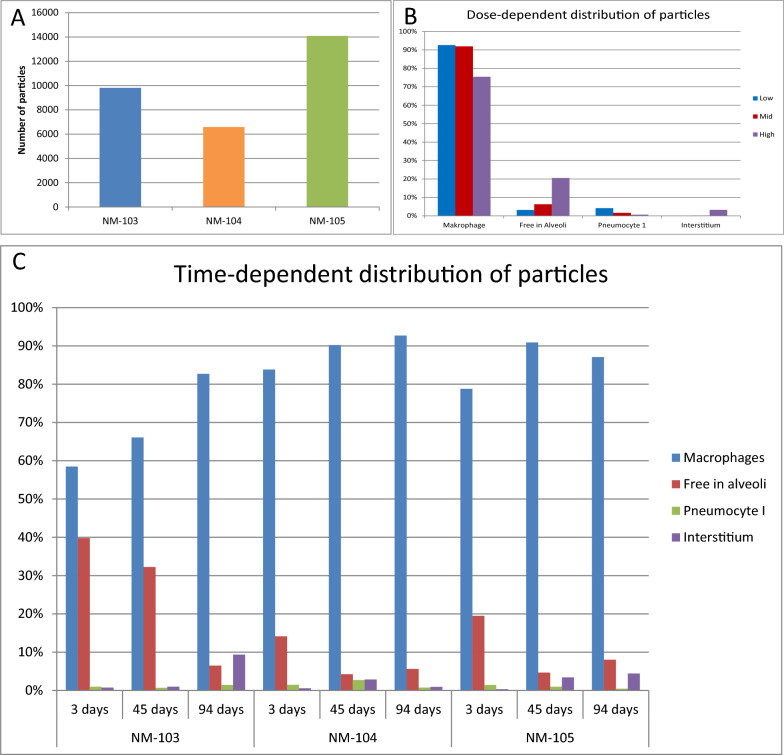
Results of the transmission electron microscopy analysis of the lung. (**A**) Number of particles detected for the different TiO_2_ (**B**) Distribution of particles dependent on the different exposure concentration **(C)** Distribution of particles dependent on the post exposure time

The total number of counted particles at all exposure-free time points and exposure concentrations of the different treatment groups (NM-103, NM-104, and MN-105) were 9805, 6587, and 14,079, respectively (Fig. 9). In general, particles were found predominantly intracytoplasmatic in intraalveolar macrophages, to a lesser extent free in the alveoli, in the lung interstitium, and intracytoplasmatic in type I pneumocytes. However, the proportional distribution of the particles varied with exposure concentration, time and treatment. Increasing the exposure concentration from low to high led to a greater number of free particles within the alveoli and in the lung interstitium, whereas the proportional distribution of alveolar macrophages and type 1 pneumocytes decreased (Fig. 9). In contrast, during the exposure free period, there was an increase in the number of particles within intraalveolar macrophages and the lung interstitium but a decrease in the number of particles within the alveoli and type 1 pneumocytes (Fig. 9). Overall, more particles were found in the NM-105-treated groups than in the NM-104-treated groups, and compared with the NM-104- and NM-105-treated groups, the NM-103-treated groups presented a greater percentage of free particles within the alveoli and a lower percentage of alveolar macrophages (Fig. 9).

## Discussion

Three different titanium dioxide nanoparticles encoded in the European nanomaterial repository, NM-103, NM-104 and NM-105, differ in their crystal structure and surface properties, suggesting different toxic potential after uptake in the lungs. To investigate this, aerosols for exposure were generated via a dry dispersion technique, mimicking an exposure scenario at workplaces. Wistar rats were exposed for 28 days to three different aerosol concentrations (3, 12 and 48 mg/m^3^), which resulted in non, partial overload and complete overload in the lungs. Endpoints were examined in compliance with OECD guideline 412 with additional transmission electron microscopy and image analysis of histologic slides. Interestingly, the three TiO_2_ varieties did not show pronounced differences in toxicity within the respiratory tract meaning the response in the respiratory tract to these different particles is similar at the same retained lung dose. Thus, the variation in properties with respect to the crystal structure and surface modification did not considerably alter the interactions with biological structures in the respiratory tract. However, some differences were apparent.

Due to physisorbed organic surface treatment with dimethicone, NM-103 became a hydrophobic particle, whereas functionalization with glycerol provided NM-104 with a hydrophilic surface. However, in this 4-week inhalation test, the surface modifications of these nanoparticles, both of which are rutile crystalline, revealed only small differences in their biological impact on the lungs. Using a similar approach, Warheit et al. [52] and Warheit & Brown [56] conducted an intratracheal instillation test in rats using pigmentary TiO_2_ particles with hydrophilic, hydrophobic and amphiphilic surfaces. Significantly different cellular and biochemical effects or histopathological findings in the respiratory tract were not detected.

NM-103 and NM-104, as rutile variants of TiO_2_, exhibited significantly stronger polymorphonuclear neutrophil (granulocyte) responses in the lungs than did the well-characterized mixed rutile/anatase variant NM-105, as can be concisely observed in the low- and mid-exposure concentration groups. The reason for this surprising outcome might be an alumina-related effect, as aluminum oxide is used for surface finishing for both NM-103 and NM-104 [38] and due to the additional chemical element present on the surface, NM-103 and NM-104 might exhibit a more toxic effect.

The TiO_2_ nanoparticle NM-105 used in this study consists of 80% anatase and 20% rutile, which suggests greater toxicity than both other particles, NM-103 and NM-104 [49, 53]. In contrast, slightly greater toxicity was observed in the NM-104-treated rats, as shown by the BAL analysis, which was also reflected by a longer retention half-time in the low- and mid-exposure concentration groups of NM-104. However, NM-105 presented a longer retention half-time in the high-exposure concentration group, which might also indicate a greater disturbance of clearance functions of the lung [33].

Electron microscopy revealed that animals, which inhaled NM-103, showed a greater percentage of particles free within the alveoli and a lower percentage within alveolar macrophages than did those in the NM-104 and NM-105 groups, which was not further substantiated by histologic examination. However, electron microscopy should be considered more sensitive. The greater number of free particles in NM-103 might be due to its hydrophobicity. Upon inhalation, the particles interact with the pulmonary surfactant. Regardless of their different physicochemical properties, particles are immediately coated with a biomolecular corona that consists of both lipids and proteins [22]. However, the structure and molecular conformation of the corona, especially the relative abundance of the surfactant-associated proteins SP-A, SP-B, and SP-C, depend on the hydrophobicity of the pristine NPs [22]. This might have affected the recognition of the particle by the macrophages, leading to less phagocytosis and more free NM-103 particles.

For all three different TiO_2_ nanoparticles (NM-103, NM-104 and NM-105), a general shift in the proportional distribution from free particles in alveoli to intracellular particles in alveolar macrophages with increasing exposure concentrations and during the exposure-free period was also observed in the histopathological investigation. This shift is also accompanied by a greater accumulation and aggregation rate of particle-laden macrophages at the terminal bronchial junction, which reflects the attempt to clear the particle-laden macrophages via the bronchi via mucociliary clearance [15]. Interestingly, in the high-exposure groups (48 mg/m^3^), this effect was less evident than that in the mid-exposure groups (12 mg/m^3^), indicating strong impairment of macrophage movement and ongoing inflammation in the high-exposure groups, in which macrophages were destroyed, as shown by the histologic investigation of the extracellular material. By using the evaluation scheme described for this study, we were able to elaborate those differences. The destruction of macrophages sets the particles free into the alveoli again and attracts granulocytes into the alveoli. Therefore, an increased number of cells other than macrophages was observed in the BALF analysis, and the image analysis of the cytospots did not reveal an obvious increase in the number of particle-laden cells between the mid and high-exposure groups. The destruction of highly loaded macrophages could also explain why the percentage of cells with high particle loads did not increase between the high- and mid-exposure groups. A burst of highly loaded macrophages has recently been described following a 90-day whole body rat inhalation study of anatase TiO_2_ with an exposure concentration 6.3, 12.5, 25, and 50 mg/m^3^ [59] . Macrophage accumulation and aggregation, as well as extracellular material and granulocyte infiltration, have recently also been described in another publication, in which 14 different inhalation studies were reviewed histologically via a similar histological examination scheme [56]. This highlights that the changes observed in the present study are compatible with the reactions observed after inhalation of poorly soluble low-toxicity particles in rats [56]. Like the macrophage aggregation observed in this study, which is also partially accompanied by alveolar epithelial type 2 cell hyperplasia, particle-laden macrophage agglomerations accompanied by reactive alveolar epithelial type 2 cell hyperplasia were described by [59]. The higher severity grade of these lesions observed in the study by [59] is most likely due to the longer exposure time of 13 weeks compared with the 4-week exposure time in the present study.

During the exposure-free periods of 45 and 94 days, increased amounts of particles in the lung interstitium were observed via electron microscopy and histology, revealing the movement of particles from the alveolar region into the lung interstitium over time, especially in the higher exposure groups. This phenomenon has been described previously and represents, compared with humans, a minor aspect of particle distribution in the rat lung [6, 15, 54]. Particle movement has also been observed in bronchus-associated lymphoid tissue (BALT) in the lung and in the lung-associated lymph nodes (LALN) via histology and image analysis of tissue slides, as described previously [15], [56]. The BALT and LALN showed during the exposure-free time increased numbers of macrophage aggregation, in addition to single particle-laden macrophages. Macrophage aggregations in lymphoid tissue are formed when macrophages cannot completely degrade phagocytized test particles [59], such as TiO_2_ particles. Additionally, toxicokinetic analysis of the lungs (particulate and soluble TiO_2_) and remote organs (liver and brain) revealed a small solubility effect under physiological conditions, and overall, the translocation to the liver and brain was negligible. No further development of histologically described macrophage aggregation into granulomatous inflammation was observed for any of the three types of TiO_2_ nanoparticles used in this study. The development of granulomatous inflammation has been described for other, more toxic particles [56]. This again highlights the fact that the different TiO_2_ nanoparticles used are poorly soluble, low-toxicity particles. However, in the mid (12 mg/m^3^) and high (48 mg/m^3^) exposure concentrations, adverse reactions were observed in all three TiO_2_ groups. These reactions included histologically observed alveolar extracellular material, which was interpreted to be cellular remnants of macrophages, and granulocyte infiltration, which was also observed in the BALF. These reactions revealed the ongoing inflammatory process in the mid and high-exposure concentration groups, which was still observable after 94 days of exposure free period.

The lung burdens in the low dose groups of NM-103, NM-104 and NM-105 at day 3 post-exposure amounted to 0.36, 0.44 and 0.48 mg/lung, respectively. Conversion of mass dose to specific surface dose results in 200, 267 and 200 cm^2^/lung, respectively. Comparison to the data presented by [48] reveals a good conformity. The surface data are exactly within the range of 200–300 cm^2^/lung concluded by the authors as an approximate threshold for non-induction of pulmonary inflammation.

The other treatment-related finding was eosinophilic globules within the nasal epithelium. This change was slightly greater in the NM-105-treated animals, especially those in the high-exposure group. It is observed in the otherwise normal epithelium of controls and can increase in incidence and severity in control rodents with age [19, 25]. The toxicological relevance of this change is not completely known, but it is thought to be indicative of slight local irritation or a defensive response [19], [57]).

## Conclusion

Since adverse reactions were observed at mid (12 mg/m^3^) and high (48 mg/m^3^) exposure concentrations in all three TiO_2_ groups, the no observed adverse effect concentration (NOAEC) was 3 mg/m^3^ for all three test items. The intended non, partial overload and complete overload conditions in the low, mid and high exposure groups could be achieved. The TiO_2_ nanoparticles, which have various surface chemistries and crystalline structures, did not show pronounced differences in toxicity. Thus, the variation in properties with respect to the crystal structure and surface modification did not considerably alter the interactions with biological structures in the respiratory tract, and the main driving force for toxicity is the particle effect and not the surface properties. However, based on the induction of polymorphonuclear neutrophil influx and it´s significant difference in the mid-exposure groups indicating adverse toxic effects the ranking was NM-104 > NM-103 > NM-105.

## Supplementary Information

Additional file 1.

Additional file 2.

Additional file 3.

Additional file 4.

Additional file 5.

Additional file 6.

Additional file 7.

Additional file 8.

Additional file 9.

## Data Availability

The data is partially available in the report from the German Federal Institute for Occupational Safety and Health (BAuA) under Grant F2246. https://www.baua.de/DE/Forschung/Forschungsprojekte/f2246.

## Abbreviations

BAL: BAL bronchoalveolar lavage
BALF: Bronchoalveolar lavage fluid
BALT: Bronchus-associated lymphoid tissue
BAUA: Bundesanstalt für Arbeitsschutz und Arbeitsmedizin (German federal institute for occupational safety and health)
ICP‒MS: Ion-coupled plasma–mass spectrometry
LALN: Lung-associated lymph node
LDH: Lactic dehydrogenase
MPPD: Multiple-path particle dosimetry model (v 0.04)
N: Number
NP: Nanoparticle
ROI: Reactive oxygen intermediate
TK: Toxicokinetic
