# Supplementary material for: Comparative toxicity study of three surface-modified titanium dioxide nanoparticles following subacute inhalation
Source: Part Fibre Toxicol. 2025 Feb 24;22:5. doi: 10.1186/s12989-025-00620-1 (PMC11849269; doi:10.1186/s12989-025-00620-1)
Supplement: Supplementary file 1 — Additional file 1. [file 12989_2025_620_MOESM1_ESM.docx]

Supplementary file 1

Overview of number of animals used for the different investigations.

| Number of animals | Other investigations | Left lung lobe | Right lung lobes |
| --- | --- | --- | --- |
| Negative control: 18  (6 animals for each exposure-free period time point)  NM-103: 54  NM-104: 54 NM-105: 54 (6 animals for each exposure concentration and exposure-free period time point)  **In total 180** | - Clinical observations (twice per day) - body weight (weekly) - macroscopy of organs upon necropsy | Bronchoalveolar lavage (BAL)  (LDH, β-Glu, total protein, ROS*)* | Chemical analytics (particle retention in lungs and in exemplary organs such as liver and brain): Lungs were processed by low temperature plasma ashing and the soluble and  insoluble moiety (the latter isolated by filtration) were separately analyzed. |
|  |  |  |  |
| Negative control: 18  (6 animals for each exposure-free period time point)  NM-103: 54  NM-104: 54 NM-105: 54 (6 animals for each exposure concentration and exposure-free period time point)  **In total 180** | - Clinical observations (twice per day) - body weight (weekly) - macroscopy of organs upon necropsy - organ weights - Histopathology (additional to lung) | Histopathology | Transmission electron microscopy: Localization  of nanoparticles in lungs; |
|  |  |  |  |
| Total number of animals: 360 |  |  |  |

Histopathology/Transmission electron microscopy analysis was done on the same animals (left lung lobe: histopathology; right lung lobes: transmission electron microscopy)

Chemical analysis/ Bronchoalveolar lavage (BAL) was done on the same animals (left lung lobe: BAL; right lung lobes: chemical analysis)
