# Supplementary material for: Comparative toxicity study of three surface-modified titanium dioxide nanoparticles following subacute inhalation
Source: Part Fibre Toxicol. 2025 Feb 24;22:5. doi: 10.1186/s12989-025-00620-1 (PMC11849269; doi:10.1186/s12989-025-00620-1)
Supplement: Supplementary file 2 — Additional file 2. [file 12989_2025_620_MOESM2_ESM.docx]

Alveolar extracellular material – residuals of inhaled test item that was deposited within the alveolar lumina. It appeared as small fine granular particles. The material was often accompanied by or consisted mainly of eosinophilic material (cellular remnants of macrophages).

Grade 1: minimal, in one or two alveoli

Grade 2: up to 40% of alveoli affected

Grade 3: approximately 40-60% of alveoli affected

Grade 4: approximately 80-90 % of alveoli affected

Grade 5: all alveoli affected

Alveolar granulocytes infiltration – isolated granulocytes within alveolar lumina

Grade 1: minimal, in one or two alveoli

Grade 2: up to 40% of alveoli affected

Grade 3: approximately 40-60% of alveoli affected

Grade 4: approximately 80-90 % of alveoli affected

Grade 5: all alveoli affected

Alveolar particles free – extracellular free TiO_2_ particles within alveolar lumina

Grade 1: minimal, in one or two alveoli

Grade 2: up to 40% of alveoli affected

Grade 3: approximately 40-60% of alveoli affected

Grade 4: approximately 80-90 % of alveoli affected

Grade 5: all alveoli affected

Perivascular mononuclear cell/granulocytes infiltration – consisting of mononuclear cells (mainly lymphocytes) or granulocytes forming a perivascular cuff.

Grade 1: < 3 vessels affected

Grade 2: < 6 vessels affected

Grade 3: < 15 vessels affected

Grade 4: < 30 vessels affected

Grade 5: > 30 vessels affected

Interstitial mononuclear cell/macrophages – consisting of mononuclear cells (mainly lymphocytes) or macrophages mainly at the terminal junction/sacs.

Grade 1: < 3 terminal sacs affected

Grade 2: < 6 terminal sacs affected

Grade 3: < 15 terminal sacs affected

Grade 4: < 30 terminal sacs affected

Grade 5: > 30 terminal sacs affected

Alveolar macrophages – isolated reactive macrophages containing inhaled material in cytoplasm within alveolar lumina (alveolar Histiocytosis)

Grade 1: < 10% of alveoli affected

Grade 2: < 25 of alveoli affected

Grade 3: < 50 of alveoli affected

Grade 4: < 75 of alveoli affected

Grade 5: > 75 of alveoli affected

Macrophage accumulation - macrophages forming loose groups within alveolar lumina, whereby cells are separated (counted from 10 cells per group onwards)

Grade 1: < 5% of alveoli affected

Grade 2: < 10 of alveoli affected

Grade 3: < 20 of alveoli affected

Grade 4: < 50 of alveoli affected

Grade 5: > 50 of alveoli affected

Macrophage aggregation - macrophages forming aggregates within alveolar lumina, whereby cells are in close contact

Grade 1: < 5% of alveoli affected

Grade 2: < 10 of alveoli affected

Grade 3: < 20 of alveoli affected

Grade 4: < 50 of alveoli affected

Grade 5: > 50 of alveoli affected

Granuloma (alveolar/bronchiolar or lymph node) – focal organized accumulation of histiocytes/macrophages at alveolar/bronchiolar junctions or within lymph nodes

Grade 1: < 5% of alveoli/bronchioli or lymph node affected

Grade 2: < 10% of alveoli/bronchiole or lymph node affected

Grade 3: < 20% of alveoli/bronchioli or lymph node affected

Grade 4: < 30% of alveoli/bronchiole or lymph node affected

Grade 5: > 30% alveoli/bronchioli or lymph node affected

Cholesterol granuloma – intraalveolar granuloma formed around cholesterol clefts

Grade 1: < 5% of alveoli affected

Grade 2: < 10% of alveoli affected

Grade 3: < 20% of alveoli affected

Grade 4: < 30% of alveoli affected

Grade 5: > 30% alveoli affected

Interstitial inflammation - Septal and interstitial accumulations of perivascular and peri-bronchiolar mononuclear inflammatory cells and more rarely contribute by granulocytic infiltration at the terminal sacs.

Grade 1: < 3 terminal sacs affected

Grade 2: < 6 terminal sacs affected

Grade 3: < 15 terminal sacs affected

Grade 4: < 30 terminal sacs affected

Grade 5: > 30 terminal sacs affected

Alveolitis, acute – focally limited acute inflammation within alveoli

Grade 1: < 5% of alveoli affected

Grade 2: < 10% of alveoli affected

Grade 3: < 20% of alveoli affected

Grade 4: < 30% of alveoli affected

Grade 5: > 30% alveoli affected

Fibrogenesis - increases in septal or interstitial thickness resulting from edema or inflammation without substantial fiber cross-linking. If associate only with minimal inflammatory infiltration considered to be fully reversible.

Grade 1: one focus minimally affected

Grade 2: several foci minimally affected

Grade 3: all foci minimally affected

Grade 4: several foci moderately affected

Grade 5: several foci severely enlarged

Fibrosis - observable increase in amount or abnormal location of collagen in lung parenchyma, resulting in disruption of the normal lung architecture. Occurrence in alveolar septa, interstitium, and pleura. Formation of distinct collagen bands.

Grade 1: < 3% of tissue affected

Grade 2: < 6% of tissue affected

Grade 3: < 15% of tissue affected

Grade 4: < 30% of tissue affected

Grade 5: > 30% of tissue affected

BALT hyperplasia – enlargement of BALT (Bronchus Associated Lymphoid Tissue) by increased number of lymphocytes.

Grade 1: one focus minimally affected

Grade 2: several foci minimally affected

Grade 3: all foci minimally affected

Grade 4: several foci moderately affected

Grade 5: several foci severely enlarged

BALT single macrophages isolated or loose groups of reactive macrophages containing inhaled material in cytoplasm.

Grade 1: up to 3 macrophages

Grade 2: 4-6 macrophages

Grade 3: 7-10 macrophages

Grade 4: 10-15 macrophages

Grade 5: >15 macrophages

BALT macrophage aggregation – accumulation of reactive histiocytes/macrophages within BALT

Grade 1: one focus minimally affected

Grade 2: several foci minimally affected

Grade 3: all foci minimally affected

Grade 4: several foci moderately affected

Grade 5: several foci severely enlarged

BALT granulomatosis infammation – accumulation of reactive histiocytes/macrophages within BALT

Grade 1: one focus minimally affected

Grade 2: several foci minimally affected

Grade 3: all foci minimally affected

Grade 4: several foci moderately affected

Grade 5: several foci severely enlarged

Hyperplasia alveolar epithelial cells (AEC) 2 – Focal or multifocal areas of increased cellularity, whereby the bronchiolo-alveolar architecture is still detectable and AEC 2 cells are dominant.

Grade 1: one focus minimally affected

Grade 2: several foci minimally affected

Grade 3: all foci minimally affected

Grade 4: several foci moderately affected

Grade 5: several foci severely enlarged

Bronchiolar hyperplasia – Focal or multifocal areas of increased cellularity,

whereby the bronchiolo-alveolar architecture is still detectable and ciliated epithelial cells are dominant and mostly single layered.

Grade 1: one focus minimally affected

Grade 2: several foci minimally affected

Grade 3: all foci minimally affected

Grade 4: several foci moderately affected

Grade 5: several foci severely enlarged

Lipoproteinosis – appearance of dense homogeneous eosinophilic material within alveoli

Grade 1: minimal, in one or two alveoli

Grade 2: up to 40% of alveoli affected

Grade 3: approximately 40-60% of alveoli affected

Grade 4: approximately 80-90 % of alveoli affected

Grade 5: all alveoli affected

Multinucleated giant cell – occurrence of multinucleated giant cells

Grade 1: < 3 multinucleated giant cell presence

Grade 2: < 6 multinucleated giant cell presence

Grade 3: < 15 multinucleated giant cell presence

Grade 4: < 30 multinucleated giant cell presence

Grade 5: > 30 multinucleated giant cell presence

Necrosis – necrotic areas within granuloma or macrophage aggregation

Grade 1: one focus minimally affected

Grade 2: several foci minimally affected

Grade 3: all foci minimally affected

Grade 4: several foci moderately affected

Grade 5: several foci severely enlarged

LALN single macrophages isolated or loose groups of reactive macrophages containing inhaled material in cytoplasm.

Grade 1: up to 3 macrophages

Grade 2: 4-6 macrophages

Grade 3: 7-10 macrophages

Grade 4: 10-15 macrophages

Grade 5: >15 macrophages

Lymph node, macrophage aggregation – accumulation of reactive histiocytes/macrophages within lymph node

Grade 1: one focus minimally affected

Grade 2: several foci minimally affected

Grade 3: all foci minimally affected

Grade 4: several foci moderately affected

Grade 5: several foci severely enlarged
