# Supplementary material for: Comparative toxicity study of three surface-modified titanium dioxide nanoparticles following subacute inhalation
Source: Part Fibre Toxicol. 2025 Feb 24;22:5. doi: 10.1186/s12989-025-00620-1 (PMC11849269; doi:10.1186/s12989-025-00620-1)
Supplement: Supplementary file 3 — Additional file 3. [file 12989_2025_620_MOESM3_ESM.pdf]

Differential cell count in the BALF after 28 days of exposure followed by 3 days of exposure free time

TABLE OF DIFFERENTIAL CELL COUNT (%)   3 days after end of exposure

Wistar Han			LEUKOCYTE CONC. [cells/ml]	MACRO-PHAGES [%]	PMNs [%]	LYMPHO-CYTES [%]
Time	Group					
Day 3 after exposure	Clean Air Control	Mean	92083	97.6	1.1	1.3
		Std	41696	1.1	1.0	1.3
		N	6	6.0	6.0	6.0
	NM-103 low	Mean	111875	#87.3	#10.5	2.2
		Std	17226	11.6	10.9	1.3
		N	6	6	6	6
	NM-103 mid	Mean	°°°*376875	°°°***59.1	°°°***37.8	3.0
		Std	65573	6.5	5.6	2.1
		N	6	6	6	6
	NM-103 high	Mean	***1146667	***46.1	***52.2	1.7
		Std	158609	8.7	8.5	1.0
		N	6	6	6	6
	NM-104 low	Mean	115000	##85.1	##12.7	2.2
		Std	33736	4.5	3.7	1.6
		N	6	6	6	6
	NM-104 mid	Mean	°°°***518750	°°°***51.3	°°°***44.8	°**3.9
		Std	102482	9.4	10.1	1.3
		N	6	6	6	6
	NM-104 high	Mean	***926667	***41.6	***56.5	1.9
		Std	341814	8.9	8.5	0.6
		N	6	6	6	6
	NM-105 low	Mean	90208	97.6	1.7	0.8
		Std	27652	1.2	1.1	0.8
		N	6	6	6	6
	NM-105 mid	Mean	°207500	°°°**77.1	°°*20.5	2.4
		Std	86869	11.8	10.8	1.6
		N	6	6	6	6
	NM-105 high	Mean	***993333	***55.8	***42.6	1.5
		Std	223107	21.6	21.3	0.9
		N	6	6	6	6

Dunnett's test: * - 5%; ** - 1%; *** - 0.1% significance level (all groups vs. controls)
Dunnett's test: # - 5%; ## - 1%; ### - 0.1% significance level (low dose groups vs. controls)
Dunnett's test: ° - 5%; °° - 1%; °°° - 0.1% significance level (mid dose groups vs. controls)

Differential cell count in the BALF after 28 days of exposure followed by 45 days of exposure free time

TABLE OF DIFFERENTIAL CELL COUNT (%) 45 days after end of exposure

Wistar Han Time	Group		LEUKOCYTE CONC. [cells/ml]	MACRO-PHAGES [%]	PMNs [%]	LYMPHO-CYTES [%]
Day 45 after exposure	Clean Air Control	Mean	66042	97.7	1.1	1.2
		Std	28322	1.8	0.7	1.3
		N	6	6	6	6
	NM-103 low	Mean	76667	97.4	1.7	0.9
		Std	35914	1.2	0.8	0.8
		N	6	6	6	6
	NM-103 mid	Mean	166043	°°°***68.9	°°°***29.7	1.4
		Std	125241	12.3	11.6	0.9
		N	6	6	6	6
	NM-103 high	Mean	***594167	***44.2	***54.2	1.6
		Std	182138	7.4	6.3	1.5
		N	6	6	6	6
	NM-104 low	Mean	72292	###93.2	###5.5	1.3
		Std	33536	2.2	2.3	1.0
		N	6	6	6	6
	NM-104 mid	Mean	°219167	°°°***61.5	°°°***34.7	°*3.8
		Std	124151	9.7	8.6	2.1
		N	6	6	6	6
	NM-104 high	Mean	***540208	***50.0	***47.9	2.0
		Std	211513	7.3	6.3	1.7
		N	6	6	6	6
	NM-105 low	Mean	103542	96.8	2.3	0.8
		Std	52389	1.3	1.3	1.1
		N	6	6	6	6
	NM-105 mid	Mean	126667	°°**78.5	°°***20.1	1.5
		Std	34230	5.5	5.1	1.3
		N	6	6	6	6
	NM-105 high	Mean	***457083	***58.0	***40.3	1.6
		Std	243590	6.1	5.2	1.4
		N	6	6	6	6

Dunnett's test: * - 5%; ** - 1%; *** - 0.1% significance level (all groups vs. controls)
Dunnett's test: # - 5%; ## - 1%; ### - 0.1% significance level (low dose groups vs. controls)
Dunnett's test: ° - 5%; °° - 1%; °°° - 0.1% significance level (mid dose groups vs. controls)

Differential cell count in the BALF after 28 days of exposure followed by 94 days of exposure free time

TABLE OF DIFFERENTIAL CELL COUNT (%) 94 days after end exposure

Wistar Han Time	Group		LEUKOCYTE CONC. [cells/ml]	MACRO-PHAGES [%]	PMNs [%]	LYMPHO-CYTES [%]
Day 94 after exposure	Clean Air Control	Mean	83958	98.8	0.7	0.5
		Std	17238	0.8	0.6	0.3
		N	6	6	6	6
	NM-103 low	Mean	122500	96.1	2.9	1.0
		Std	63161	3.3	3.6	0.8
		N	6	6	6	6
	NM-103 mid	Mean	66458	°°°***76.0	°°**18.5	°°°***5.5
		Std	14522	10.8	11.7	2.2
		N	6	6	6	6
	NM-103 high	Mean	***615000	***49.1	***47.8	3.0
		Std	479041	12.1	11.8	1.8
		N	6	6	6	6
	NM-104 low	Mean	95625	##91.4	#7.4	1.2
		Std	53856	5.8	5.9	0.7
		N	6	6	6	6
	NM-104 mid	Mean	94167	°°°***67.8	°°°***28.0	°°**4.2
		Std	28358	8.2	8.1	2.7
		N	6	6	6	6
	NM-104 high	Mean	334792	***51.5	***43.8	***4.8
		Std	154443	8.3	8.0	2.4
		N	6	6	6	6
	NM-105 low	Mean	104375	98.4	1.2	0.4
		Std	74619	1.9	1.9	0.3
		N	6	6	6	6
	NM-105 mid	Mean	74792	°°**81.0	°°*15.9	3.1
		Std	21757	4.8	5.7	1.8
		N	6	6	6	6
	NM-105 high	Mean	**429167	***46.2	***50.8	3.0
		Std	153571	11.8	11.1	1.8
		N	6	6	6	6

Dunnett's test: * - 5%; ** - 1%; *** - 0.1% significance level (all groups vs. controls)
Dunnett's test: # - 5%; ## - 1%; ### - 0.1% significance level (low dose groups vs. controls)
Dunnett's test: ° - 5%; °° - 1%; °°° - 0.1% significance level (mid dose groups vs. controls)
