# Supplementary material for: Comparative toxicity study of three surface-modified titanium dioxide nanoparticles following subacute inhalation
Source: Part Fibre Toxicol. 2025 Feb 24;22:5. doi: 10.1186/s12989-025-00620-1 (PMC11849269; doi:10.1186/s12989-025-00620-1)
Supplement: Supplementary file 4 — Additional file 4. [file 12989_2025_620_MOESM4_ESM.pdf]

Secretion of reactive oxygen intermediates (ROI) of 2 x 10^5 alveolar lavage cells in reactive light units (RLU) at day 3, 45 and 94 of the exposure free period

	Day 3		Day 45		Day 94	
	Medium	Zymosan	Medium	Zymosan	Medium	Zymosan
**Clean Air Control**						
Mean	1.953E+06	**5.471E+06**	2.212E+06	**6.129E+06**	2.990E+06	**4.022E+06**
SD	± 6.01E+05	± 1.78E+06	± 6.10E+05	± 1.26E+06	± 1.13E+06	± 1.05E+06
N	6	6	6	6	6	6
**NM-103, low**						
Mean	1.760E+06	**5.944E+06**	1.894E+06	**5.643E+06**	2.007E+06	**4.776E+06**
SD	± 4.91E+05	± 1.43E+06	± 7.01E+05	± 1.64E+06	± 1.11E+06	± 1.13E+06
N	5	5	5	5	5	5
**NM-103, mid**						
Mean	3.996E+05	**7.427E+06**	7.334E+05	**6.743E+06**	1.442E+06	**4.015E+06**
SD	± 8.42E+04	± 1.75E+06	± 3.01E+05	± 1.10E+06	± 6.70E+05	± 7.89E+05
N	5	5	5	5	5	5
**NM-103, high**						
Mean	1.435E+05	**7.587E+06**	1.355E+05	**6.168E+06**	3.733E+05	**4.107E+06**
SD	± 6.59E+04	± 3.03E+06	± 7.28E+04	± 2.11E+06	± 2.65E+05	± 1.67E+06
N	5	5	5	5	5	5
**NM-104, low**						
Mean	1.363E+06	**5.510E+06**	1.448E+06	**5.902E+06**	2.436E+06	**4.466E+06**
SD	± 3.74E+05	± 1.44E+06	± 2.21E+05	± 7.72E+05	± 1.66E+06	± 1.18E+06
N	5	5	5	5	5	5
**NM-104, mid**						
Mean	3.165E+05	****8.694E+06**	4.988E+05	**7.207E+06**	9.253E+05	**5.081E+06**
SD	± 1.15E+05	± 1.57E+06	± 1.84E+05	± 4.97E+05	± 3.90E+05	± 1.08E+06
N	5	5	5	5	5	5
**NM-104, high**						
Mean	***1.845E+05	***7.772E+06**	1.857E+05	**6.698E+06**	3.589E+05	**4.239E+06**
SD	± 9.41E+04	± 1.28E+06	± 2.51E+04	± 1.44E+06	± 2.02E+05	± 1.62E+06
N	5	5	5	5	5	5
**NM-105, low**						
Mean	1.830E+06	**6.515E+06**	2.481E+06	**7.244E+06**	3.237E+06	***5.927E+06**
SD	± 6.13E+05	± 1.54E+06	± 1.01E+06	± 8.46E+05	± 1.22E+06	± 1.57E+06
N	5	5	5	5	5	5
**NM-105, mid**						
Mean	1.294E+06	**6.926E+06**	1.023E+06	**6.492E+06**	1.518E+06	**3.955E+06**
SD	± 7.35E+05	± 1.76E+06	± 2.55E+05	± 1.23E+06	± 6.88E+05	± 1.10E+06
N	5	5	5	5	5	5
**NM-105, high**						
Mean	1.822E+05	**6.973E+06**	2.707E+05	**6.854E+06**	4.376E+05	**5.076E+06**
SD	± 9.21E+04	± 2.15E+06	± 1.19E+05	± 1.86E+06	± 1.88E+05	± 1.21E+06
N	5	5	5	5	5	5

Student's t-test: * - 5%; ** - 1%; *** - 0.1% significance level (treated groups vs. clean air controls)
