# Supplementary material for: Comparative toxicity study of three surface-modified titanium dioxide nanoparticles following subacute inhalation
Source: Part Fibre Toxicol. 2025 Feb 24;22:5. doi: 10.1186/s12989-025-00620-1 (PMC11849269; doi:10.1186/s12989-025-00620-1)
Supplement: Supplementary file 5 — Additional file 5. [file 12989_2025_620_MOESM5_ESM.pdf]

## Overview table on retention results

| PARTICULATE | Mean | ASD | RSD | Mean | ASD | RSD | Mean | ASD | RSD |
|---|---|---|---|---|---|---|---|---|---|
| | (µg/lung) | | (%) | (µg/lung) | | (%) | (µg/lung) | | (%) |
| | Day 3 | | | Day 45 | | | Day 94 | | |
| Clean Air Control | < 5 | | | < 5 | | | < 5 | | |
| | | | | | | | | | |
| NM-103, low | 358 | 80 | 22.3 | 179 | 44 | 24.4 | 122 | 30 | 24.3 |
| NM-103, mid | 1625 | 248 | 15.3 | 1530 | 199 | 13.0 | 1107 | 166 | 15.0 |
| NM-103, high | 7081 | 918 | 13.0 | 7664 | 987 | 12.9 | 6028 | 510 | 8.5 |
| | | | | | | | | | |
| NM-104, low | 436 | 41 | 9.5 | 370 | 147 | 39.8 | 209 | 43 | 20.6 |
| NM-104, mid | 1698 | 189 | 11.1 | 1674 | 145 | 8.6 | 1344 | 92 | 6.8 |
| NM-104, high | 3782 | 507 | 13.4 | 3928 | 479 | 12.2 | 3113 | 211 | 6.8 |
| | | | | | | | | | |
| NM-105, low | 477 | 140 | 29.4 | 255 | 93 | 36.3 | 121 | 27 | 22.5 |
| NM-105, mid | 1819 | 180 | 9.9 | 1806 | 356 | 19.7 | 1345 | 141 | 10.5 |
| NM-105, high | 5879 | 594 | 10.1 | 6679 | 622 | 9.3 | 5212 | 844 | 16.2 |
| | | | | | | | | | |
| SOLUBLE | Mean | ASD | RSD | Mean | ASD | RSD | Mean | ASD | RSD |
| | (µg/lung) | | (%) | (µg/lung) | | (%) | (µg/lung) | | (%) |
| | Day 3 | | | Day 45 | | | Day 94 | | |
| Clean Air Control | < 2.5 | | | < 2.5 | | | < 2.5 | | |
| | | | | | | | | | |
| NM-103, low | 8.5 | 2.3 | 27.6 | 5.8 | 2.1 | 36.7 | 4.8 | 0.6 | 11.8 |
| NM-103, mid | 9.6 | 6.3 | 66.2 | 10.7 | 2.6 | 24.6 | 13.2 | 5.7 | 42.9 |
| NM-103, high | 40.0 | 16.6 | 41.5 | 37.0 | 8.1 | 21.8 | 16.9 | 3.2 | 19.0 |
| | | | | | | | | | |
| NM-104, low | 12.1 | 5.2 | 43.3 | 7.2 | 1.3 | 17.8 | 11.4 | 3.2 | 27.9 |
| NM-104, mid | 11.2 | 5.2 | 46.7 | 17.4 | 4.7 | 26.8 | 9.8 | 3.8 | 38.9 |
| NM-104, high | 34.6 | 5.1 | 14.8 | 16.1 | 3.7 | 22.9 | 17.9 | 8.5 | 47.8 |
| | | | | | | | | | |
| NM-105, low | 21.8 | 9.3 | 42.6 | 7.9 | 3.6 | 46.0 | 4.3 | 1.4 | 31.8 |
| NM-105, mid | 17.4 | 10.6 | 60.7 | 39.9 | 18.6 | 46.6 | 18.1 | 2.6 | 14.3 |
| NM-105, high | 54.0 | 6.5 | 12.0 | 51.8 | 12.2 | 23.6 | 44.4 | 4.4 | 9.8 |

ASD Absolute standard deviation
RSD Relative standard deviation

Processing of organ samples
Samples were cut into smaller pieces and oxidized using low-temperature plasma ashing. The remaining ash was put into 25 ml water (Milli-Q) and shaken until homogeneity. After a further 30-min period the particle suspension was filtrated using a 0.2 µm Whatman-Nuclepore filter (pore filter); then the filter was rinsed with 25 ml additional water. Recoveries: Plasma ashing - Ionic Ti: 103-105%; Filtration - Ionic Ti: 96%; Chemical analysis - QC standards: 101%; NBS SRM 349: 102%

Juxtaposition of particulate vs. soluble moieties of the test items
(lungs, in µg/lung)

| | Day 3 | | | | Day 45 | | | | Day 94 | | | |
|---|---|---|---|---|---|---|---|---|---|---|---|---|
| | P | S | T | S | P | S | T | S | P | S | T | S |
| | | | | (%) | | | | (%) | | | | (%) |
| **Control** | < 5 | < 2.5 | | | < 5 | < 2.5 | | | < 5 | < 2.5 | | |
| | | | | | | | | | | | | |
| **NM-103, low** | 358 | 8.5 | 366 | 2.3 | 179 | 5.8 | 185 | 3.1 | 122 | 4.8 | 135 | 3.5 |
| **NM-103, mid** | 1625 | 9.6 | 1635 | 0.6 | 1530 | 10.7 | 1541 | 0.7 | 1107 | 13.2 | 1120 | 1.2 |
| **NM-103, high** | 7081 | 40.0 | 7121 | 0.6 | 7664 | 37.0 | 7701 | 0.5 | 6028 | 16.9 | 6045 | 0.3 |
| | | | | | | | | | | | | |
| **NM-104, low** | 436 | 12.1 | 448 | 2.7 | 370 | 7.2 | 377 | 1.9 | 209 | 11.4 | 220 | 5.5 |
| **NM-104, mid** | 1698 | 11.2 | 1710 | 0.7 | 1674 | 17.4 | 1691 | 1.0 | 1344 | 9.8 | | 0.7 |
| **NM-104, high** | 3782 | 34.6 | 3817 | 0.9 | 3928 | 16.1 | 3944 | 0.4 | 2860 | 17.9 | 2878 | 0.6 |
| | | | | | | | | | | | | |
| **NM-105, low** | 477 | 21.8 | 499 | 4.4 | 255 | 7.9 | 262 | 3.0 | 121 | 4.3 | 125 | 3.5 |
| **NM-105, mid** | 1819 | 17.4 | 1836 | 0.9 | 1806 | 39.9 | 1846 | 2.2 | 1345 | 18.1 | 1363 | 1.3 |
| **NM-105, high** | 5879 | 54.0 | 5933 | 0.9 | 6679 | 51.8 | 6731 | 0.8 | 5527 | 44.4 | 5531 | 0.8 |

| | |
|---|---|
| P | Particulate moiety |
| S | Soluble moiety |
| T | Total: Particulate + soluble moiety |
| S (%) | Soluble moiety in percent of total mass |
