# Supplementary material for: Comparative toxicity study of three surface-modified titanium dioxide nanoparticles following subacute inhalation
Source: Part Fibre Toxicol. 2025 Feb 24;22:5. doi: 10.1186/s12989-025-00620-1 (PMC11849269; doi:10.1186/s12989-025-00620-1)
Supplement: Supplementary file 6 — Additional file 6. [file 12989_2025_620_MOESM6_ESM.pdf]

Retention Measurements (individual data with means)

Retention of particulate TiO_2 in liver

LIVER									
	**Mean**	**ASD**	**RSD**	**Mean**	**ASD**	**RSD**	**Mean**	**ASD**	**RSD**
	**(µg/tissue)**		**(%)**	**(µg/tissue)**		**(%)**	**(µg/tissue)**		**(%)**
	Day 3			Day 45			Day 94		
Clean Air Control	< 5			< 5			< 5		
	< 5			< 5			< 5		
	< 5			< 5			< 5		
	< 5			< 5			< 5		
	< 5			< 5			< 5		
	< 5			< 5			< 5		
	**< 5**			**< 5**			**< 5**		
NM-103, low	< 5			< 5			< 5		
	< 5			< 5			< 5		
	< 5			< 5			< 5		
	< 5			< 5			< 5		
	< 5			< 5			< 5		
	< 5			< 5			< 5		
	**< 5**			**< 5**			**< 5**		
NM-103, mid									
	< 5			< 5			< 5		
	< 5			< 5			< 5		
	< 5			< 5			< 5		
	< 5			< 5			< 5		
	< 5			< 5			< 5		
	< 5			< 5			< 5		
	**< 5**			**< 5**			**< 5**		
NM-103, high									
	< 5			< 5			< 5		
	< 5			< 5			< 5		
	< 5			< 5			< 5		
	< 5			< 5			< 5		
	< 5			< 5			< 5		
	5.4	0.4	6.9	< 5			< 5		
	**< 5**			**< 5**			**< 5**		

Individual data given in normal font
Group mean data given in **bold font**

Retention Measurements (individual data with means)

Retention of particulate TiO_2 in liver

LIVER									
	**Mean**	**ASD**	**RSD**	**Mean**	**ASD**	**RSD**	**Mean**	**ASD**	**RSD**
	**(µg/tissue)**		**(%)**	**(µg/tissue)**		**(%)**	**(µg/tissue)**		**(%)**
	Day 3			Day 45			Day 94		
Clean Air Control	< 5			< 5			< 5		
	< 5			< 5			< 5		
	< 5			< 5			< 5		
	< 5			< 5			< 5		
	< 5			< 5			< 5		
	< 5			< 5			< 5		
	**< 5**			**< 5**			**< 5**		
NM-104, low	< 5			< 5			< 5		
	< 5			< 5			< 5		
	< 5			< 5			< 5		
	< 5			< 5			< 5		
	< 5			< 5			< 5		
	< 5			< 5			< 5		
	**< 5**			**< 5**			**< 5**		
NM-104, mid	< 5			< 5			13.6	1.3	9.8
	< 5			< 5			< 5		
	< 5			< 5			< 5		
	< 5			< 5			< 5		
	< 5			< 5			14.2	1.0	7.3
	< 5			< 5			< 5		
	**< 5**			**< 5**					
NM-104, high	< 5			< 5			**206**	**2**	**0.9**
	< 5			< 5			< 5		
	< 5			< 5			< 5		
	< 5			< 5			< 5		
	< 5			< 5			< 5		
	< 5			< 5			**3.6**	**0.2**	**6.5**
	**< 5**			**< 5**					

Individual data given in normal font
Group mean data given in **bold font**

Retention Measurements (individual data with means)

Retention of particulate TiO_2 in liver

LIVER									
	**Mean**	**ASD**	**RSD**	**Mean**	**ASD**	**RSD**	**Mean**	**ASD**	**RSD**
	**(µg/tissue)**		**(%)**	**(µg/tissue)**		**(%)**	**(µg/tissue)**		**(%)**
	Day 3			Day 45			Day 94		
Clean Air Control	< 5			< 5			< 5		
	< 5			< 5			< 5		
	< 5			< 5			< 5		
	< 5			< 5			< 5		
	< 5			< 5			< 5		
	< 5			< 5			< 5		
	**< 5**			**< 5**			**< 5**		
NM-105, low	< 5			< 5			< 5		
	< 5			< 5			< 5		
	< 5			< 5			< 5		
	< 5			< 5			< 5		
	< 5			< 5			< 5		
	< 5			< 5			< 5		
	**< 5**			**< 5**			**< 5**		
NM-105, mid	< 5			< 5			< 5		
	< 5			< 5			< 5		
	< 5			< 5			< 5		
	< 5			< 5			< 5		
	< 5			< 5			< 5		
	< 5			< 5			< 5		
	**< 5**			**< 5**			**< 5**		
NM-105, high	< 5			< 5			**66.5/3.4**	**0.4/0.1**	**0.7/3.0**
	< 5			< 5			< 5		
	< 5			< 5			< 5		
	< 5			< 5			< 5		
	< 5			16.3	0.5	3.1	< 5		
	< 5			< 5			< 5		
	**< 5**								

Individual data given in normal font
Group mean data given in **bold font**

Retention Measurements (individual data with means)

Retention of particulate TiO_2 in brain

BRAIN									
	**Mean**	**ASD**	**RSD**	**Mean**	**ASD**	**RSD**	**Mean**	**ASD**	**RSD**
	**(µg/tissue)**		**(%)**	**(µg/tissue)**		**(%)**	**(µg/tissue)**		**(%)**
	Day 3			Day 45			Day 94		
Clean Air Control	< 2.5			< 2.5			< 2.5		
	< 2.5			< 2.5			< 2.5		
	< 2.5			< 2.5			< 2.5		
	< 2.5			< 2.5			< 2.5		
	< 2.5			< 2.5			< 2.5		
	< 2.5			< 2.5			< 2.5		
	**< 2.5**			**< 2.5**			**< 2.5**		
NM-103, low	< 2.5			< 2.5			< 2.5		
	< 2.5			< 2.5			< 2.5		
	< 2.5			< 2.5			< 2.5		
	< 2.5			< 2.5			< 2.5		
	< 2.5			< 2.5			< 2.5		
	< 2.5			< 2.5			< 2.5		
	**< 2.5**			**< 2.5**			**< 2.5**		
NM-103, mid									
	< 2.5			< 2.5			< 2.5		
	< 2.5			< 2.5			< 2.5		
	< 2.5			< 2.5			< 2.5		
	< 2.5			< 2.5			< 2.5		
	< 2.5			< 2.5			< 2.5		
	< 2.5			< 2.5			< 2.5		
	**< 2.5**			**< 2.5**			**< 2.5**		
NM-103, high									
	< 2.5			< 2.5			< 2.5		
	< 2.5			< 2.5			< 2.5		
	< 2.5			< 2.5			< 2.5		
	< 2.5			< 2.5			< 2.5		
	< 2.5			< 2.5			< 2.5		
	< 2.5			< 2.5			< 2.5		
	**< 2.5**			**< 2.5**			**< 2.5**		

Individual data given in normal font
Group mean data given in **bold font**

Retention Measurements (individual data with means)

Retention of particulate TiO_2 in brain

BRAIN									
	**Mean**	**ASD**	**RSD**	**Mean**	**ASD**	**RSD**	**Mean**	**ASD**	**RSD**
	**(µg/tissue)**		**(%)**	**(µg/tissue)**		**(%)**	**(µg/tissue)**		**(%)**
	Day 3			Day 45			Day 94		
Clean Air Control	< 2.5			< 2.5			< 2.5		
	< 2.5			< 2.5			< 2.5		
	< 2.5			< 2.5			< 2.5		
	< 2.5			< 2.5			< 2.5		
	< 2.5			< 2.5			< 2.5		
	< 2.5			< 2.5			< 2.5		
	**< 2.5**			**< 2.5**			**< 2.5**		
NM-104, low	< 2.5			< 2.5			< 2.5		
	< 2.5			< 2.5			< 2.5		
	< 2.5			< 2.5			< 2.5		
	3.3	0.3	8.9	< 2.5			< 2.5		
	< 2.5			< 2.5			< 2.5		
	< 2.5			< 2.5			< 2.5		
	**< 2.5**			**< 2.5**			**< 2.5**		
NM-104, mid	< 2.5			< 2.5			< 2.5		
	< 2.5			< 2.5			< 2.5		
	< 2.5			< 2.5			< 2.5		
	< 2.5			< 2.5			< 2.5		
	< 2.5			< 2.5			< 2.5		
	< 2.5			< 2.5			< 2.5		
	**< 2.5**			**< 2.5**			**< 2.5**		
NM-104, high	< 2.5			< 2.5			< 2.5		
	< 2.5			< 2.5			< 2.5		
	< 2.5			< 2.5			< 2.5		
	< 2.5			< 2.5			< 2.5		
	< 2.5			< 2.5			< 2.5		
	< 2.5			< 2.5			< 2.5		
	**< 2.5**			**< 2.5**			**< 2.5**		

Individual data given in normal font
Group mean data given in **bold font**

Retention Measurements (individual data with means)

Retention of particulate TiO_2 in brain

BRAIN									
	**Mean**	**ASD**	**RSD**	**Mean**	**ASD**	**RSD**	**Mean**	**ASD**	**RSD**
	**(µg/tissue)**		**(%)**	**(µg/tissue)**		**(%)**	**(µg/tissue)**		**(%)**
	Day 3			Day 45			Day 94		
Clean Air Control	< 2.5			< 2.5			< 2.5		
	< 2.5			< 2.5			< 2.5		
	< 2.5			< 2.5			< 2.5		
	< 2.5			< 2.5			< 2.5		
	< 2.5			< 2.5			< 2.5		
	< 2.5			< 2.5			< 2.5		
	**< 2.5**			**< 2.5**			**< 2.5**		
NM-105, low	< 2.5			< 2.5			< 2.5		
	< 2.5			< 2.5			< 2.5		
	< 2.5			< 2.5			< 2.5		
	< 2.5			< 2.5			< 2.5		
	< 2.5			< 2.5			< 2.5		
	< 2.5			< 2.5			< 2.5		
	**< 2.5**			**< 2.5**			**< 2.5**		
NM-105, mid	< 2.5			< 2.5			< 2.5		
	< 2.5			< 2.5			< 2.5		
	< 2.5			< 2.5			< 2.5		
	< 2.5			< 2.5			< 2.5		
	< 2.5			< 2.5			< 2.5		
	< 2.5			< 2.5			< 2.5		
	**< 2.5**			**< 2.5**			**< 2.5**		
NM-105, high	< 2.5			< 2.5			< 2.5		
	< 2.5			< 2.5			< 2.5		
	< 2.5			< 2.5			< 2.5		
	< 2.5			< 2.5			< 2.5		
	< 2.5			< 2.5			< 2.5		
	< 2.5			< 2.5			< 2.5		
	**< 2.5**			**< 2.5**			**< 2.5**		

Individual data given in normal font
Group mean data given in **bold font**
