# Supplementary material for: Comparative toxicity study of three surface-modified titanium dioxide nanoparticles following subacute inhalation
Source: Part Fibre Toxicol. 2025 Feb 24;22:5. doi: 10.1186/s12989-025-00620-1 (PMC11849269; doi:10.1186/s12989-025-00620-1)
Supplement: Supplementary file 9 — Additional file 9. [file 12989_2025_620_MOESM9_ESM.pptx]

## Slide 1
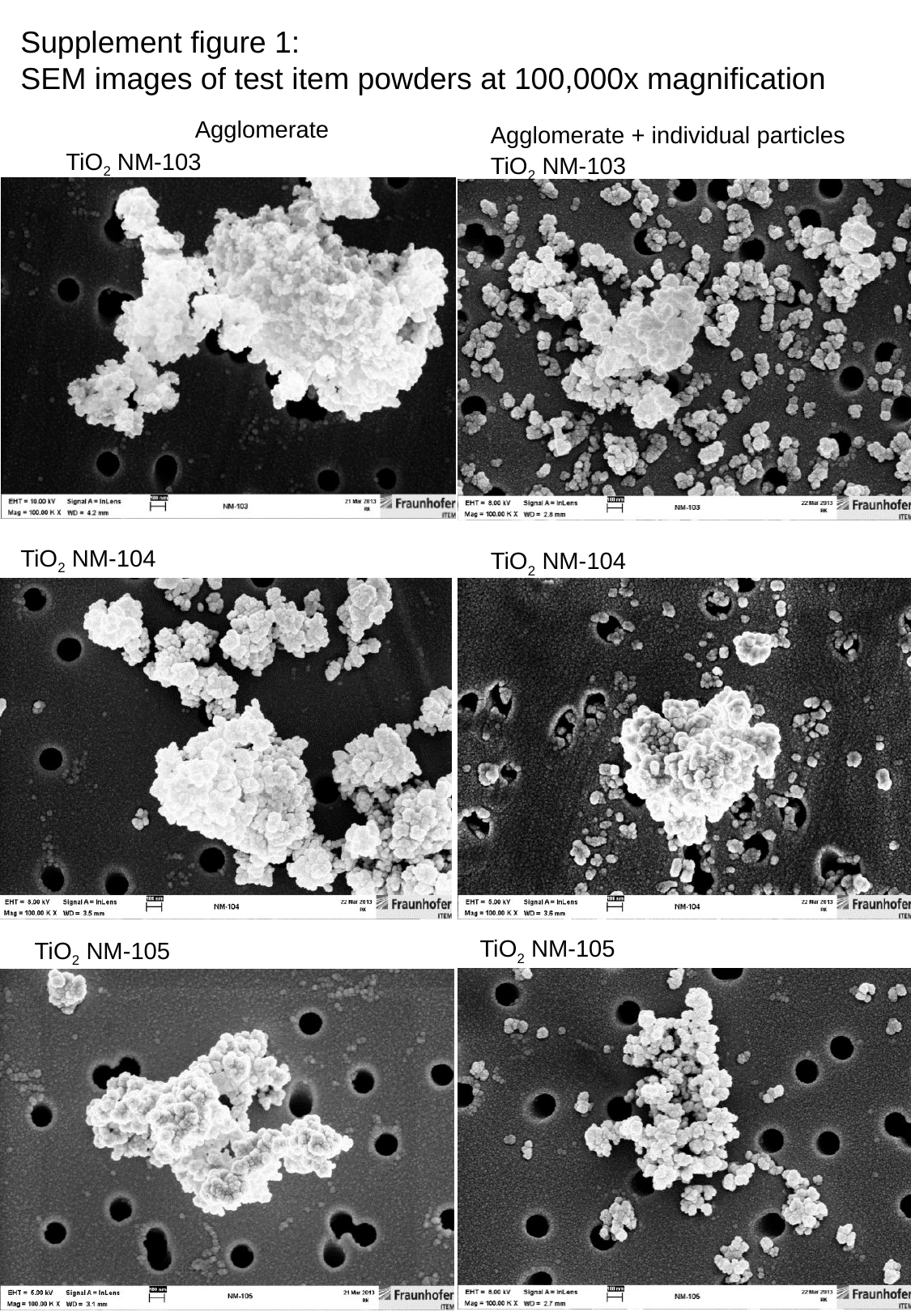

Supplement figure 1:
SEM images of test item powders at 100,000x magnification
Agglomerate
Agglomerate + individual particles
TiO2 NM-103
TiO2 NM-103
TiO2 NM-104
TiO2 NM-104
TiO2 NM-105
TiO2 NM-105

## Slide 2
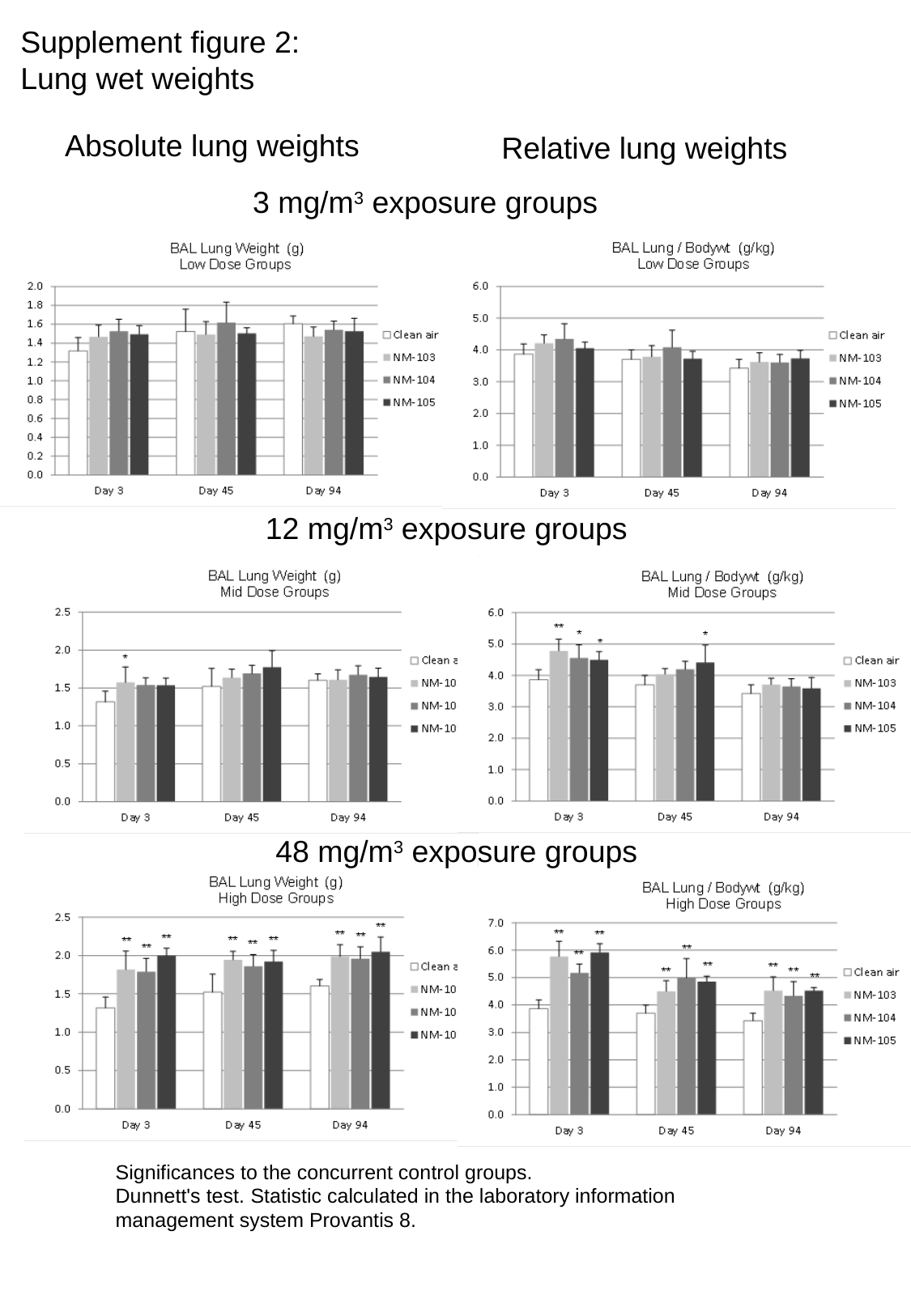

Supplement figure 2:
Lung wet weights
Absolute lung weights
Relative lung weights
3 mg/m3 exposure groups
12 mg/m3 exposure groups
48 mg/m3 exposure groups
Significances to the concurrent control groups.
Dunnett's test. Statistic calculated in the laboratory information management system Provantis 8.

## Slide 3
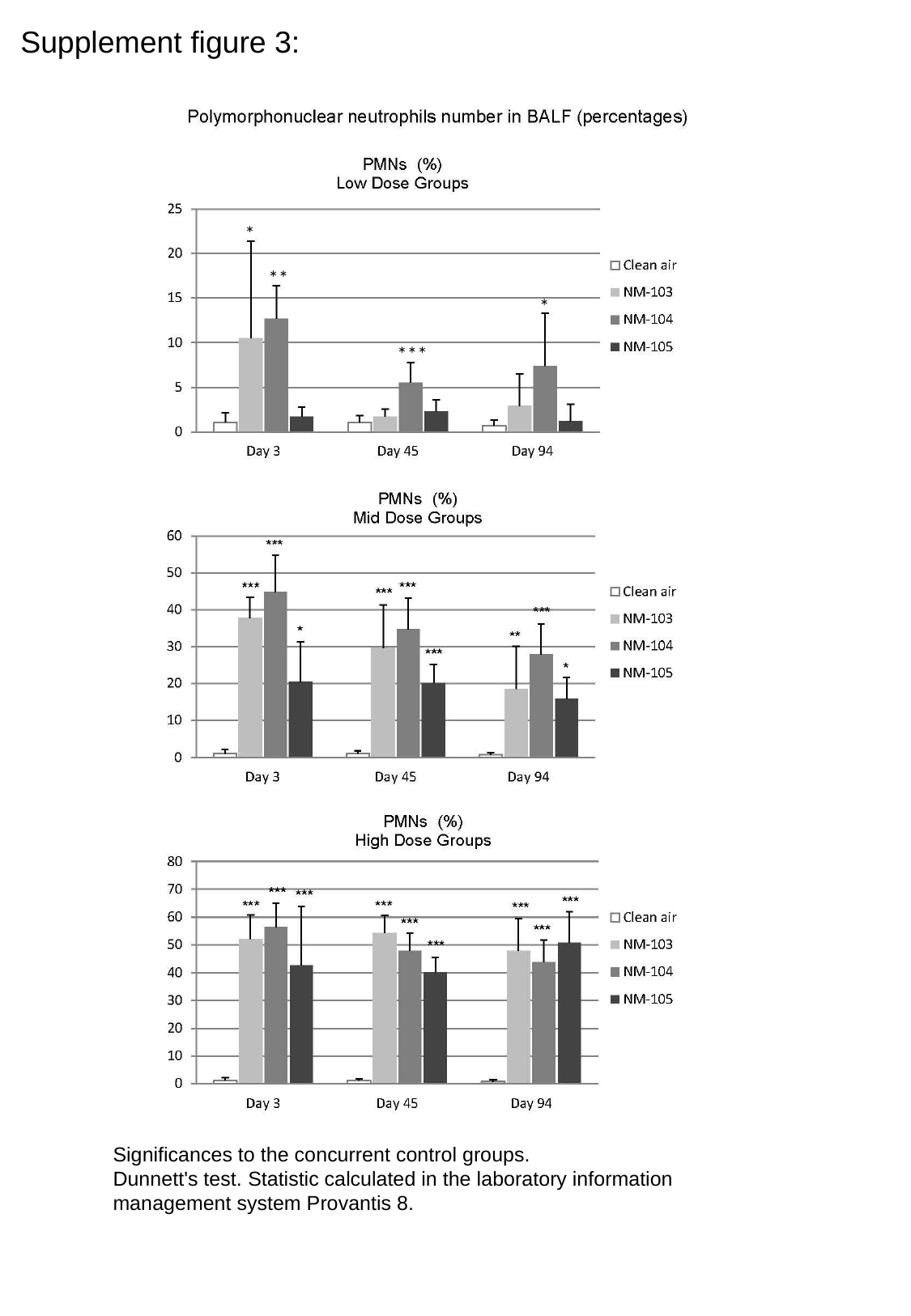

Supplement figure 3:
#
*
**
*
***
Significances to the concurrent control groups.
Dunnett's test. Statistic calculated in the laboratory information management system Provantis 8.

## Slide 4
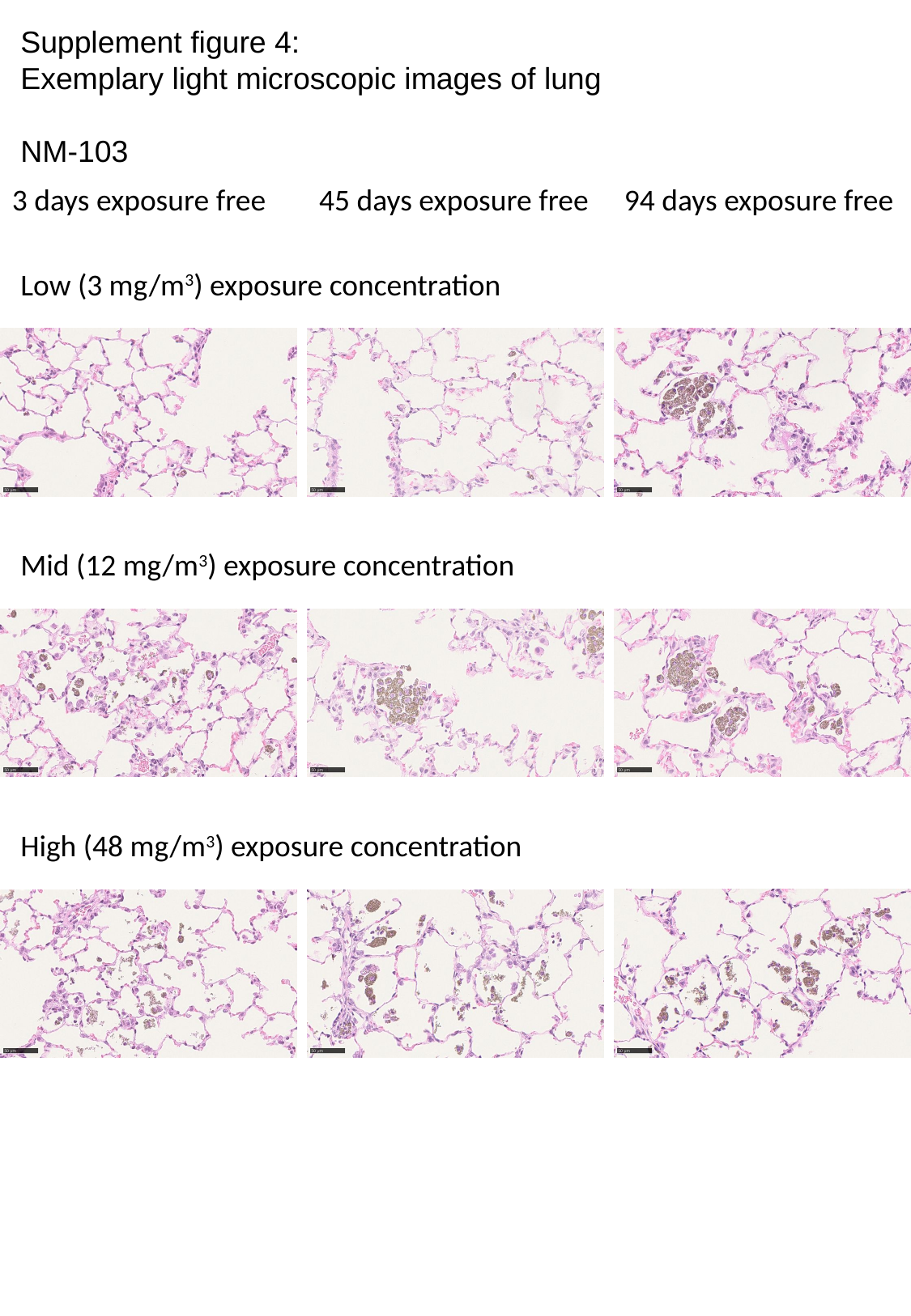

Supplement figure 4:
Exemplary light microscopic images of lung
NM-103
3 days exposure free
45 days exposure free
94 days exposure free
Low (3 mg/m3) exposure concentration
Mid (12 mg/m3) exposure concentration
High (48 mg/m3) exposure concentration

## Slide 5
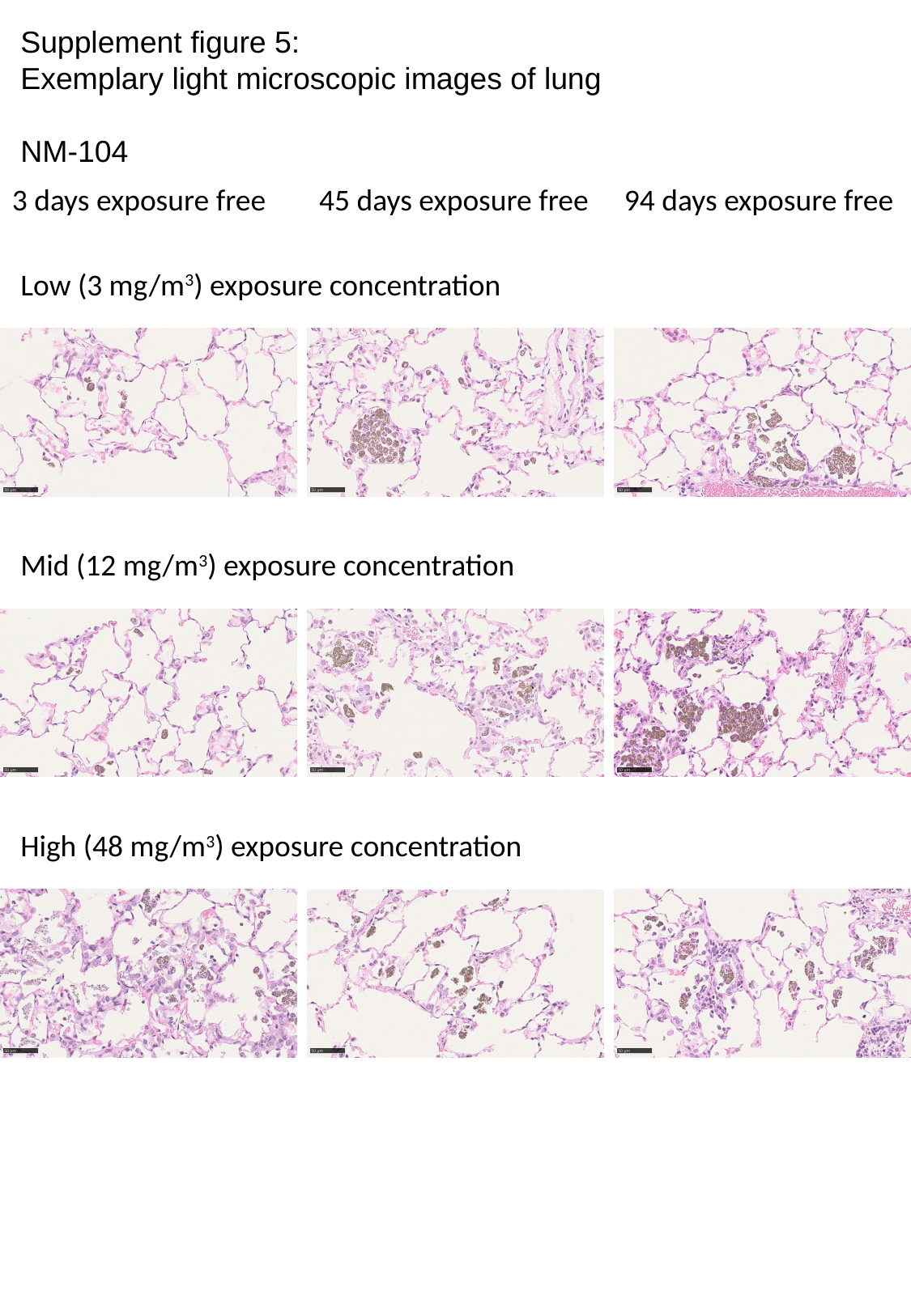

Supplement figure 5:
Exemplary light microscopic images of lung
NM-104
3 days exposure free
45 days exposure free
94 days exposure free
Low (3 mg/m3) exposure concentration
Mid (12 mg/m3) exposure concentration
High (48 mg/m3) exposure concentration

## Slide 6
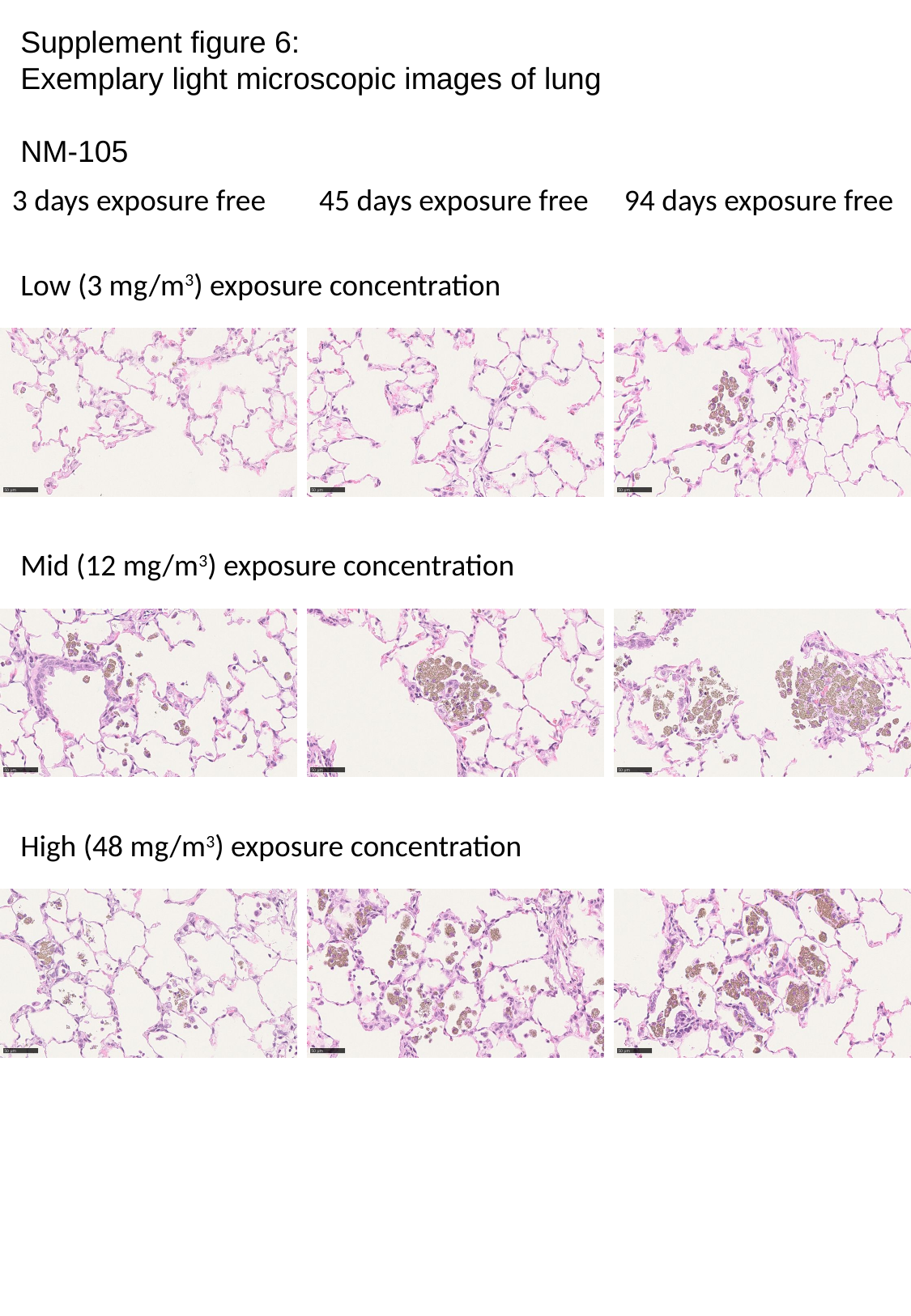

Supplement figure 6:
Exemplary light microscopic images of lung
NM-105
3 days exposure free
45 days exposure free
94 days exposure free
Low (3 mg/m3) exposure concentration
Mid (12 mg/m3) exposure concentration
High (48 mg/m3) exposure concentration

## Slide 7
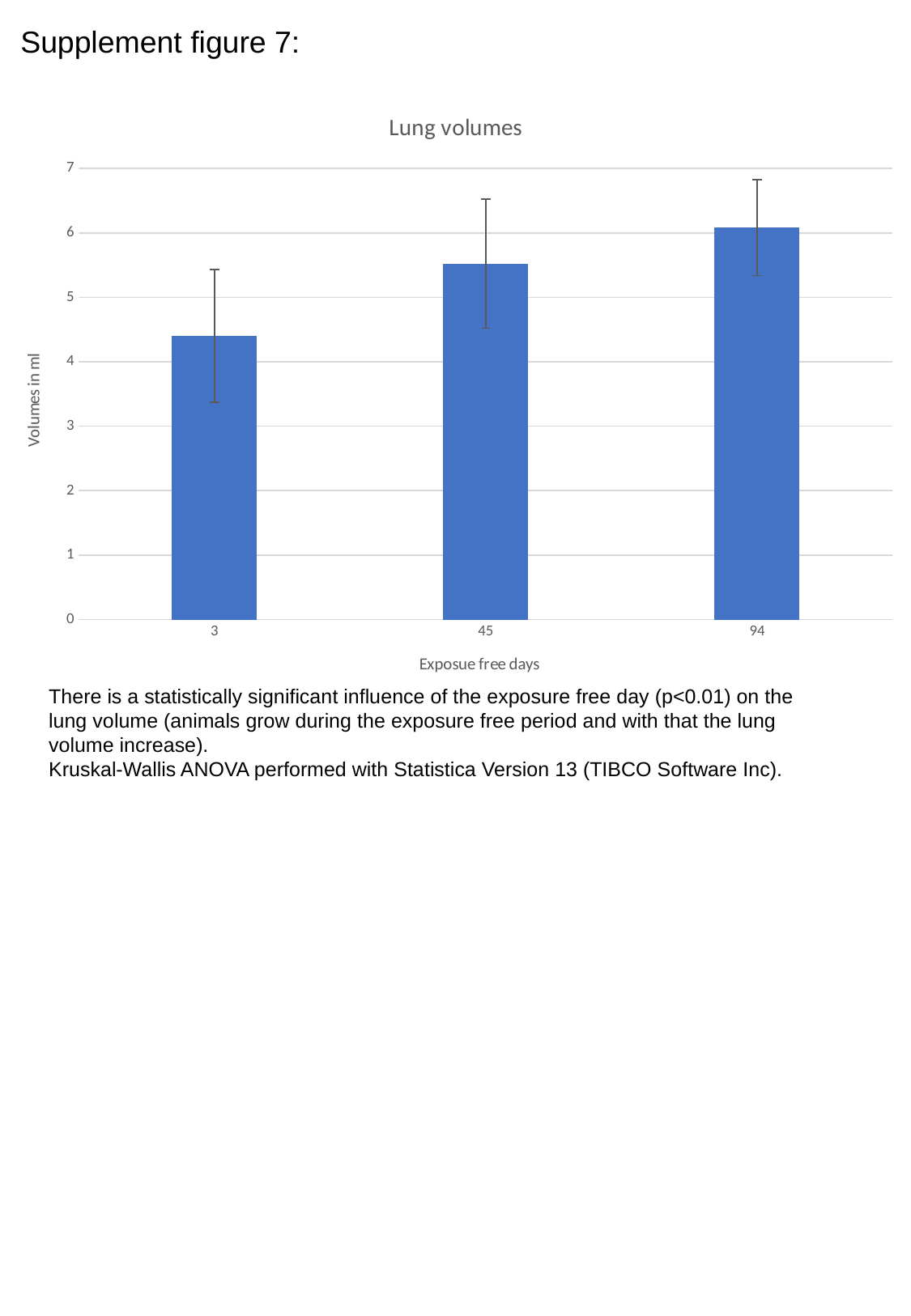

Supplement figure 7:
### Chart: Lung volumes
| Category | |
|---|---|
| 3 | 4.404 |
| 45 | 5.523333333333334 |
| 94 | 6.082500000000001 |There is a statistically significant influence of the exposure free day (p<0.01) on the lung volume (animals grow during the exposure free period and with that the lung volume increase).
Kruskal-Wallis ANOVA performed with Statistica Version 13 (TIBCO Software Inc).

## Slide 8
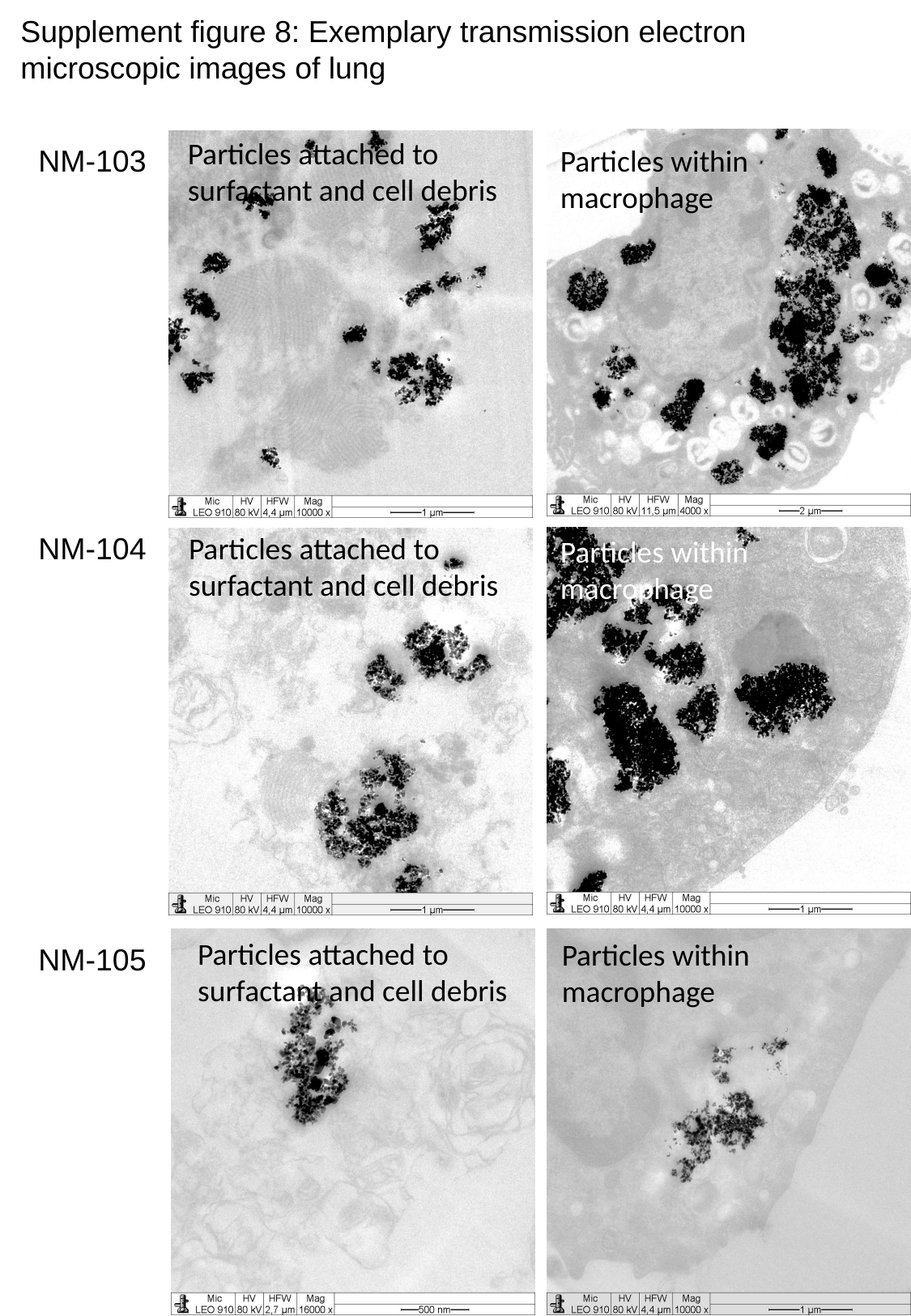

Supplement figure 8: Exemplary transmission electron microscopic images of lung
Particles attached to surfactant and cell debris
Particles within macrophage
NM-103
Particles attached to surfactant and cell debris
NM-104
Particles within macrophage
Particles attached to surfactant and cell debris
Particles within macrophage
NM-105
